# Anemonefishes: A model system for evolutionary genomics

**DOI:** 10.12688/f1000research.130752.2

**Published:** 2023-10-27

**Authors:** Marcela Herrera, Timothy Ravasi, Vincent Laudet

**Affiliations:** 1 Marine Eco-Evo-Devo Unit, Okinawa Institute of Science and Technology Graduate University, 1919-1 Tancha, Onna-son, Okinawa, 904-0495, Japan; 2 Marine Climate Change Unit, Okinawa Institute of Science and Technology Graduate University, 1919-1 Tancha, Onna-son, Okinawa, 904-0495, Japan; 3 Australian Research Council Centre of Excellence for Coral Reef Studies, James Cook University, Townsville, Queensland, 4811, Australia; 4 Marine Research Station, Institute of Cellular and Organismic Biology (ICOB), Academia Sinica, 23-10, Dah-Uen Rd, Jiau Shi I-Lan 262, Taiwan

**Keywords:** adaptive radiation, Amphiprion, chromosome-scale assembly, clownfish, genome, pigmentation, proteomics, transcriptomics

## Abstract

Anemonefishes are an iconic group of coral reef fish particularly known for their mutualistic relationship with sea anemones. This mutualism is especially intriguing as it likely prompted the rapid diversification of anemonefish. Understanding the genomic architecture underlying this process has indeed become one of the holy grails of evolutionary research in these fishes. Recently, anemonefishes have also been used as a model system to study the molecular basis of highly complex traits such as color patterning, social sex change, larval dispersal and life span. Extensive genomic resources including several high-quality reference genomes, a linkage map, and various genetic tools have indeed enabled the identification of genomic features controlling some of these fascinating attributes, but also provided insights into the molecular mechanisms underlying adaptive responses to changing environments. Here, we review the latest findings and new avenues of research that have led to this group of fish being regarded as a model for evolutionary genomics.

## 1. Introduction

**Figure 1.**
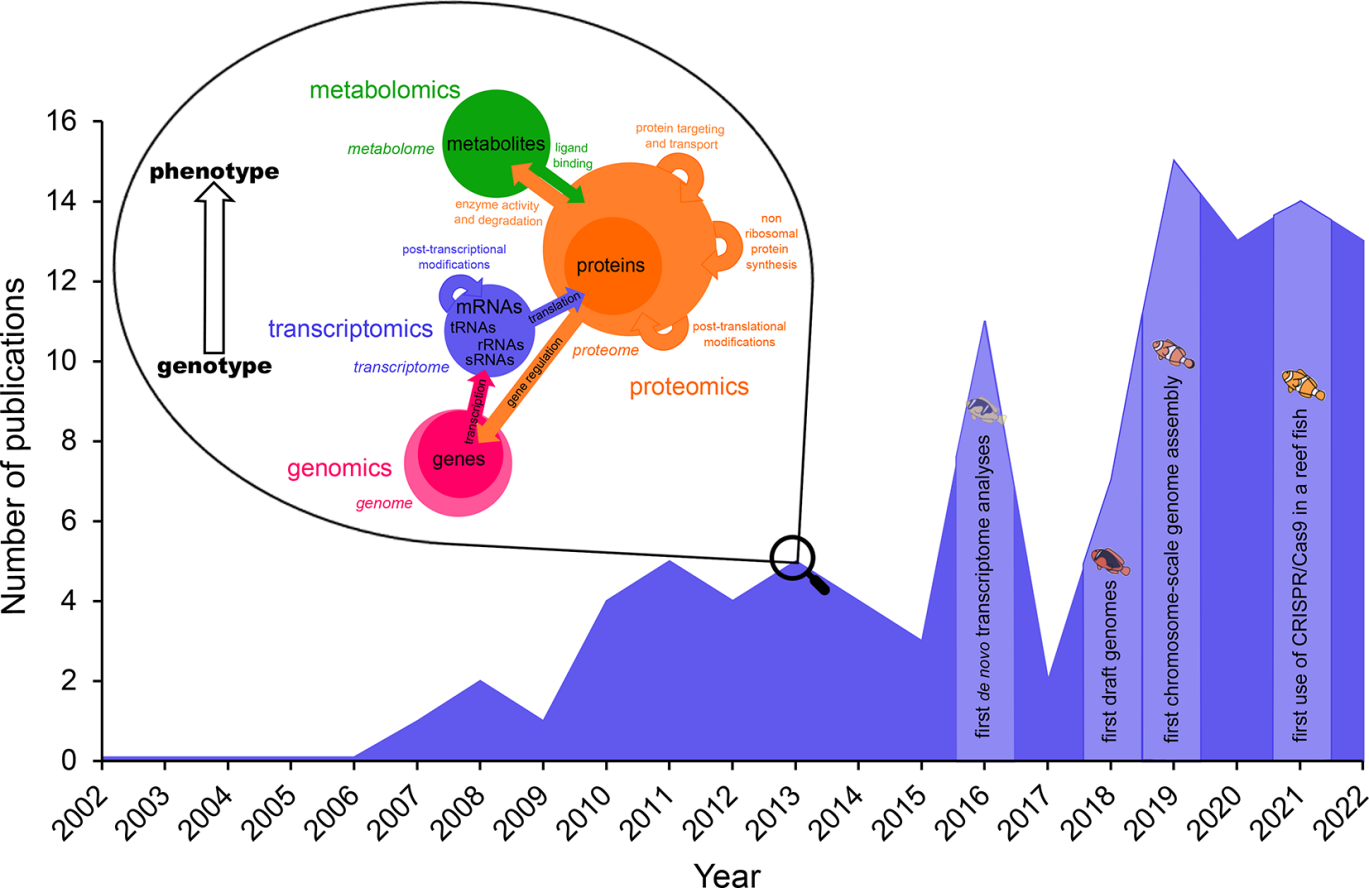
Overview of the “omics” technologies used in anemonefish research. The green circle represents metabolomics, orange -proteomics, blue -transcriptomics, and pink -genomics. Colored arrows indicate interactions between the metabolome, proteome, transcriptome, and genome and how they affect each other. Circle sizes illustrate estimated complexity (adapted from Grimm *et al*. 2021). Number of publications using “omics” tools were retrieved from the Web of Knowledge ( https://apps.webofknowledge.com/) and plotted according to year of publication. Since the early 2000’s when the first studies investigating gene expression and technological advancement of various molecular sequencing platforms, the application of “omics” tools has increased steadily and led to the achievement of milestones such as the assembly of one of the most contiguous chromosome-scale fish genomes and the successful use of CRISPR/Cas9 gene editing in a reef fish (as shown by the light areas in the plot). Keywords used to determine these studies were separated into independent variables (or) within two categories donated by (and): “gene expression or genome or transcriptome or proteome or genomics or transcriptomics or proteomics or omics” and “clownfish or anemonefish or *Amphiprion*”.

The increasing availability of genomic tools and resources is revolutionizing our understanding of the molecular basis of evolution. ^1^ Rapid advancements are being made in addressing questions such as: how does speciation occur, and how do new adaptations drive this process? Which genetic changes are responsible for morphological, physiological, and behavioral traits? How do organisms cope with rapidly changing environments? For the past decade, anemonefish have been a valuable tool for ecological and evolutionary research, but the development of molecular methods in recent years has made it possible to apply it to previously intractable problems in developmental biology, adaptive evolution, and speciation (reviewed in Refs. 2, 3). Whole-genome, transcriptome, and proteome sequencing, collectively known as “omics” tools ( Figure 1), have opened anemonefish research up to new possibilities, hypotheses, and information regarding their ecology and evolution.

The year 2019 saw the publication of the first chromosome-scale genome for an anemonefish ^4^ ( Figure 1). Of all published chromosome-level fish genomes to that date, the *Amphiprion percula* genome stood out as one of the most contiguous fish genomes with ~98% of the assembled genome ordered into chromosomes. ^4^^,^^5^ This impressive feat not only highlighted the power of modern genome sequencing but most importantly, it empowered an array of studies in anemonefish (it has been cited 51 times as of July 2023 according to Google Scholar). Furthermore, the possibility of whole mRNA sequencing propelled transcriptome studies in a variety of tissues from multiple species and life stages to examine gene expression changes in development and adaptive responses to environmental stressors (reviewed in Section 2.3). Though not specifically for anemonefish, a growing number of studies are applying proteomics in coral reef fish (see Section 2.4). Monroe and colleagues (2020) used a mass spectrometry data-independent acquisition method for proteome quantification in a non-model fish species for the first time, ^6^ a stepping stone for the application of proteomics to study many anemonefish.

Previously, “omics” methods were only used in a handful of studies, but now are one of the most common tools applied in the field and thus the primary focus of this review. Here, we describe the numerous attributes that make anemonefish an exceptional model system for studying evolutionary genomics. We then present a detailed synthesis of recent research that has provided important insights into the incredible adaptive radiation anemonefish have undergone, ^7^^–^^11^ and how their genomic architecture underlies the evolution of complex phenotypic traits such as sex change ^12^^,^^13^ and color patterning. ^14^^–^^16^ We further describe how researchers are using anemonefish as a model system to understand the genomic basis of symbiosis with giant sea anemones ^10^^,^^17^^–^^19^ and environmental plasticity. ^20^^,^^21^

## 2. Anemonefish as a model system for evolutionary biology

**Figure 2.**
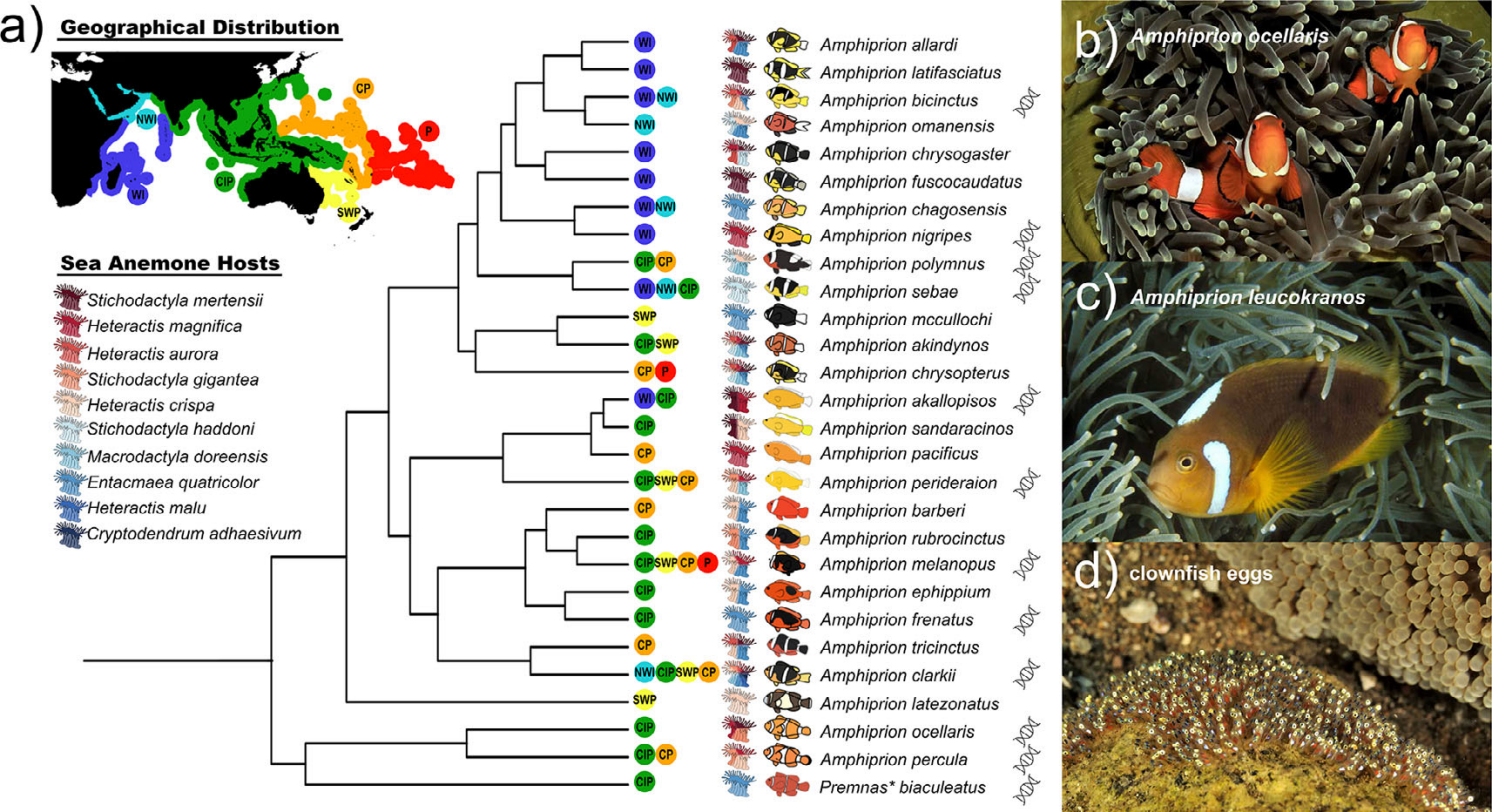
Mutualism with sea anemones triggered the adaptive radiation of anemonefish. a) Phylogeny of anemonefishes based on the 20 most informative genes (adapted from Marcionetti *et al.* 2022). Geographical distributions (light blue: NWI – North-Western Indian Ocean, dark blue: WI – Western Indian Ocean, green: CIP –Central Indo-Pacific Ocean, orange: CP – Central Pacific Ocean, yellow: SWP – South-Western Pacific Ocean, red: P – Polynesian Ocean), sea anemone hosts, and phenotypes are shown for each species. Asterisk denotes a recent revision of anemonefish phylogenetic data that suggests *Premnas biaculeatus* should be recognized as *Amphiprion.* Lastly, DNA symbol is shown next to the species for which genomes have been sequenced. b) The iconic false clownfish *Amphiprion ocellaris.* c) The white bonnet anemonefish *Amphiprion leucokranos* is a naturally occurring hybrid species found in the WI and CIP regions. d) Clownfish lay hundreds of eggs on the substrate near their host anemone. Pictures taken by Pascal Kobeh.

Before presenting the various contributions made in the field of anemonefish research, we feel it is essential to clarify the difference between the terms “anemonefish” and “clownfish” both of which are used throughout this review. Conventionally, the English name “anemonefish” has been associated with the distinctive symbiosis between these fish species and giant sea anemones, while the term “clownfish” highlights their vibrant colors and bold behavior. In line with the prevailing practice among researchers in this field, ^22^ we have opted to refer to them as “anemonefish” to acknowledge the pivotal importance of their symbiotic relationship with giant sea anemones, a key aspect that profoundly influences their biology. However, for *Amphiprion ocellaris* and *Amphiprion percula*, two closely related species forming a natural subgroup within anemonefish, we do use the term “clownfish”. There are 28 species of anemonefish ^2^ ( Figure 2a), yet the two clownfish *A. ocellaris* ( Figure 2b) and *A. percula* are perhaps the most recognizable ones, especially following the Disney movie “Finding Nemo”. ^23^ Within the more than 300 species in the family Pomacentridae (to which anemonefish belong), two genera have been previously described: *Amphiprion* and *Premnas*, the latter including only one species which is being now considered as part of *Amphiprion* (reviewed in Section 3.1) ^24^^,^^25^ ( Figure 2a). All anemonefishes are protandrous hermaphrodites (i.e., male to female transition) that live in association with sea anemones. ^26^^,^^27^ It is a mutualistic relationship in which the sea anemone provides food and shelter from predators ^26^^,^^27^ and, in return, the territorial fish protects its host from predation by attacking other animals that attempt to feed on the tentacles. ^28^ Furthermore, the fish provides supplemental nutrition source ^29^ and also increases oxygen uptake by modulating water flow among the tentacles. ^30^^,^^31^ Social groups typically consist of an adult breeding pair and several smaller (immature) juveniles ranked by size. ^32^ Basically, a large dominant female is followed by a male so that if the female is removed, the male changes sex and the largest non-breeder matures into a breeding male. ^12^ This male is also the one providing most of the parental care to the eggs by keeping them clean and well-oxygenated, another fascinating and rare feature of anemonefishes. ^2^

Living in symbiosis with 10 distantly related sea anemones ( *Cryptodendrum* spp., *Entacmaea* spp., *Heteractis* spp., *Macrodactyla* spp., and *Stichodactyla* spp.), anemonefish can be found in shallow, tropical waters of the Indo-Pacific Ocean, from Australia to the Ryukyu archipelago (Japan) and from the Red Sea and southwest coast of Africa to the Maldives and French Polynesia. There is no anemonefish in the Caribbean nor in the Eastern Pacific (e.g., Hawaii). ^27^^,^^33^ The highest diversity is in the Coral Triangle, ^27^^,^^34^ where up to nine species have been observed coexisting together. ^35^ Distribution varies greatly for each species, some are widespread (e.g., *A. clarkii*, *A. sandaracinos*), while others have a limited regional distribution (e.g., *A. bicinctus*, *A. percula*) or are even restricted to a few islands (e.g., *A. chagosensis*, *A. mccullochi*). Similarly, some species can only associate with one sea anemone (i.e., specialists), whereas other may have various possible hosts (i.e., generalists) ( Figure 2a). For example, the yellowtail clownfish *A. clarkii*, for which a chromosome-scale reference genome was recently published, ^36^ is the only species that has been observed to inhabit all 10 species of sea anemone. ^27^^,^^33^ As such, it also has the widest distribution ^33^ and temperature tolerance, ^37^ making it a robust and accessible study species. In contrast, the tomato clownfish *A. frenatus* can only be found in one species of sea anemone ( *Entacmaea quadricolor*). ^27^^,^^33^ Furthermore, hybridization in the wild has been observed and several hybrid species (such as *A. leucokranos* ( Figure 2c)) are known, ^38^^–^^41^ prompting the study of its role in anemonefish evolution. ^8^^,^^11^^,^^42^ Early studies have already characterized the ecology, behavior, and diversity of many traits of anemonefish, laying the groundwork for subsequent research using a variety of genomic tools that facilitated key discoveries across many biological fields (reviewed in Ref. 22).

### 2.1 Practical features of anemonefish for experimentation

One of the main reasons why anemonefish have become a model organism for a broad range of biological disciplines (e.g., host-microbiome interactions, ^10^^,^^17^ developmental ^20^ and phenotypic plasticity, ^43^ behavior, ^44^^,^^45^ and larval dispersal dynamics ^46^^–^^48^) is because they can be easily found in the field due to their symbiosis with giant sea anemones, but they can also complete their life cycle in captivity. ^2^^,^^49^ In contrast to adults, which are easy to observe and collect in their natural environment (as they almost never abandon their host anemone), studying wild anemonefish larvae represents a major difficulty. This has led to the development of husbandry methods and experimental protocols for “low-volume” rearing, and rearing and hatching embryos without parental care. ^49^^,^^50^ Furthermore, anemonefish can adapt to different system types and culture conditions (e.g., closed or open systems, filtered or natural seawater, different temperatures and salinities), and do not require the presence of a host anemone, which makes their maintenance easier. ^49^^,^^50^ Such rearing methods and intrinsic features of anemonefish has expanded their potential as a model organism by opening new avenues for the use of molecular tools (e.g., micro-injections ^51^^,^^52^), functional approaches, and ecotoxicological and/or pharmacological experiments (reviewed in ^2^). Crosses can be performed in the laboratory ^16^ and anemonefish can spawn every two to three weeks, typically laying between 100 and 500 eggs on the substrate near their host anemone ( Figure 2d). Further, they have a short embryonic and larval development (which have been characterized in precise detail ^53^^,^^54^) of 10–15 days (depending on the water temperature and the species), thus allowing large-scale studies. ^2^ Generation times, however, can be as long as 18 months (depending on the species), which might impose practical constraints on experimental approaches. Nevertheless, the biological traits and practical features in their breeding described above make them a growing model organism for developmental biology, ecology, and evolutionary sciences.

### 2.2 High-quality reference genomes

**Figure 3.**
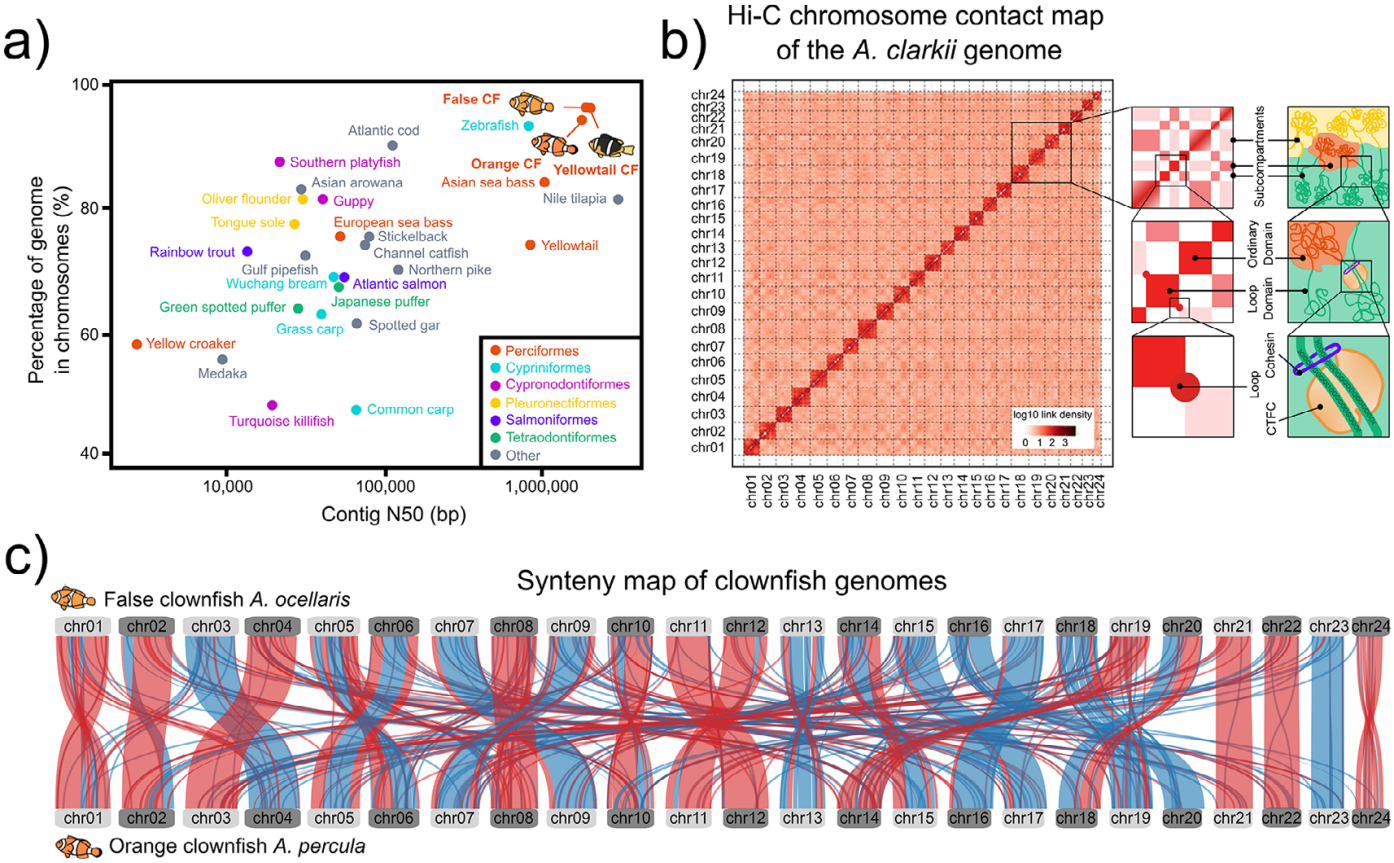
Advances in genomics of anemonefish. a) A comparison of genome contiguity for the three anemonefish chromosome-scale genomes (the false clownfish *Amphiprion ocellaris*, the orange clownfish *Amphiprion percula*, and the yellowtail clownfish *Amphiprion clarkii*) and 26 other previously published chromosome-scale fish genomes assemblies until 2019 (adapted from Lehman *et al.* 2019 and Hotaling *et al.* 2019). Data points are color-coded by order. b) Chromatin contact mapping takes advantage of the inverse relationship between proximity of nuclear DNA and genomic distance thus allowing contigs to be clustered into chromosomal groups. Here, the Hi-C heatmap of interactions between pairs of chromosomal loci (chr01-chr24) throughout the *A. clarkii* genome is shown. Interactions were drawn based on the chromatin interaction frequencies between pairs of 100 kb genomic regions (as determine by Hi-C). Darker red cells indicate stronger and more frequent interactions, which in turn imply that the two sequences are spatially close. Close-ups on the right show an overview of features revealed by Hi-C maps. Top squares show the long-range contact pattern of a locus (left) and its nuclear subcompartments (right). Middle squares show enhanced contact frequency along the diagonal (left) which indicate the presence of small domains of condensed chromatin (right). Bottom squares show peaks in the contact map (left) and the presence of loops that lie at domain boundaries and bind CTCF (right) (adapted from Rao *et al*. 2014). c) Availability of high-quality chromosome scale assemblies allow for large-scale genomic comparisons. Here, a dual synteny plot between all 24 chromosomes from *A. ocellaris* and *A. percula* shows conserved sequences within chromosomes of both species. Chromosomal rearrangements such as translocations and inversions are shown as red ribbons, whereas blue ribbons represent unchanged regions. Chromosomes in both species have been designated based on their size following and orderly arrangement from the largest to the smallest.

As significant advances in sequencing technologies have been made and genome projects become more affordable, ^55^^,^^56^ a new era of genome biology in anemonefishes has also begun. It was only until recently, in 2018, that the first draft genome assembly for an anemonefish, that of the false clownfish *A. ocellaris,* ^57^ was published. Though the coverage of this genome was low (~11×), it allowed the prediction of 27,240 high-quality protein-coding genes (96.3% of which were functionally annotated) and a genome size of 880 Mb. This was soon followed by the genomes of *A. frenatus* ^58^ and *A. percula,* ^4^ the latter of which was, until not long ago, one of the most contiguous and complete teleost fish genome assemblies currently available. ^5^ Constructing a high-quality chromosome-level assembly for a species with no previous genome-scale data was certainly a major achievement in a world dominated by sticklebacks and zebrafish genomic research ( Figure 3a). Since then, the genomes of at least nine other species ^10^^,^^36^ have been sequenced and deposited in public databases ( Figure 2a). While these resources can provide valuable insights into the molecular evolution and adaptation of common anemonefish traits, ^10^^,^^11^^,^^36^^,^^59^^,^^60^ with the exception of *A. percula* ^4^ and the recently published chromosome-scale assemblies of *A. ocellaris* ^60^ and *A. clarkii* ^36^ ( Figure 3a), most of these genomes ^10^^,^^58^ are mainly based on Illumina technology and are therefore highly fragmented. A large number of scaffolds can result in multiple gaps and mis-assemblies, which can then hinder the understanding of genomic features such as chromosome rearrangements, gene duplications, repetitive regions, and changes in regulatory sequences. ^61^^,^^62^ Thus, even if the functional content of these genomes (26,917–29,913 genes containing 92.7–94.9% of the core set of actinopterygian orthologs) is similar to that of the chromosome-level assemblies mentioned above, their lower quality might pose a challenge for certain types of analyses.

Compared to second-generation sequencing technologies, third-generation sequencing platforms such as Pacific Biosciences (also known as single-molecule, real-time (SMRT) sequencing) and Oxford Nanopore Technologies (ONT) can produce long reads (5-50 kb for PacBio sequencing and up to the current record of 2.3 Mb for Nanopore reads ^63^), thus making it possible to assemble genomes with higher contiguity and completeness. Indeed, inclusion of Nanopore reads together with Illumina data led to a 94% decrease in the number of scaffolds and an 18-fold increase in N50, and increased the genome completeness of *A. ocellaris* by an additional 16%. ^64^ Moreover, the past decade has also seen the rise of chromatin contact (Hi-C) mapping to achieve chromosome-level assemblies. ^65^ By crosslinking and fragmenting stretches of DNA that are physically close, and using short-read technology to paired-end sequence these fragments, the frequency distribution of how often two fragments of the genome interact (i.e., how physically close they are to one another) can be known and used to cluster contigs into chromosomal groups ^65^ ( Figure 3b). The contiguity of the *A. percula* assembly, the first chromosome-scale reference assembly for an anemonefish, improved dramatically following this approach. Scaffold N50 increased from 1.9 to 38.1 Mb, a more than 20-fold improvement, and over 1,000 contigs were placed into 24 chromosomal scaffolds. ^4^^,^^5^ Furthermore, the recently published chromosome-scale assembly of the yellowtail clownfish *A. clarkii* ^36^ has the highest quality and completeness (protein-coding genes encompassing 97.0% of conserved actinopterygian genes) of all published anemonefish genomes to date ^4^^,^^10^^,^^58^^,^^60^^,^^64^ and is possibly in the upper echelons of all previously published fish genomes as well ^66^^–^^71^ ( Figure 3a).

The availability of genomes for multiple species has been critical to gain insights into the evolutionary history and adaptive radiation of anemonefishes. High-quality assemblies allow for comparative genomics and molecular evolution analyses on different traits of anemonefish. For example, using the first *A. ocellaris* long-read assembled genome, ^57^ gene-editing with CRISPR/Cas9 became possible for the first time. ^51^ The recently published chromosome-scale assembly of *A. ocellaris* ^60^ revealed genomic elements conserved only in *A. ocellaris* and its sister species *A. percula* ( Figure 3c). Importantly, the authors found that these elements are close to genes implicated in various nervous system functions and distinct expression patterns in the brain, potentially highlighting the genetic toolkits involved in lineage-specific divergence and behaviors of the ocellaris/percula branch. Comparative analysis using the *A. clarkii* genome ^36^ identified higher copy numbers of the *erbb3b* gene. Notably, *erbb3b* encodes for an epidermal growth factor receptor (EGFR)-like tyrosinase kinase linked to melanophore development, suggesting then a possible link between this gene and the natural melanism polymorphism observed in *A. clarkii.* ^72^ Studying anemonefish genomes has also enabled the identification of visual opsin genes and analysis of their synteny with the *A. percula* genome. ^59^ There is evidence of two tandem duplication events involving the ultraviolet-sensitive ( *SWS1*) opsin gene, of which two functionally-coding genes ( *SWS1α* and *SWS1β*) are retained in the genomes of all anemonefishes. This is an exceptionally rare finding as most teleost fishes have lost this gene altogether. ^73^ Last but not least, the genomes published by Marcionetti and colleagues (2019) have been crucial to identify genes under positive selection. ^10^ Particularly, this study identified 17 genes at the origin of anemonefish radiation, two of which ( *versican core protein* and *protein O-GlcNAse*) have been hypothesized to remove and/or mask *N*-acetylated sugars present in the skin mucus that protect anemonefish from getting stung by the sea anemone (reviewed in Section 3.2). Thus, providing the first insights into the genetic mechanisms of clownfish mutualism with sea anemones.

### 2.3 Insights from comparative transcriptomics

The first molecular insights of anemonefish biology came well before any of the genomes now available were sequenced. Early-day research by Jones and colleagues with microsatellites shed a light on the population genetics and dispersal patterns of anemonefish in Kimbe Bay, Papua New Guinea. ^46^^,^^74^^,^^75^ Yet, it was a 2010 study using quantitative polymerase chain reaction (qPCR) to investigate the role of the aromatase *cyp19a1* gene on sex differentiation of the yellowtail clownfish *A. clarkii* ^76^ that really pioneered molecular research in anemonefish. Nowadays, applications of transcriptomics have rapidly expanded, and with it our understanding of the mechanistic underpinnings of various biological processes such as development, adaptive evolution, disease progression, and stress response. Indeed, the transcriptome is dynamic compared to the genome and is useful for dissecting the relationship between genotype and phenotype. ^77^^,^^78^ RNA-seq and qPCR are low-cost methods that can provide high-resolution data without the need for extensive genomic resources. ^78^ In anemonefish, transcriptome sequencing and qPCR experiments have provided insights into gene expression changes during development ^12^^,^^15^^,^^76^^,^^79^^–^^83^ and adaptive responses to variations in the environment. ^21^^,^^84^^–^^86^ Numerous efforts have contributed to the paramount abundance of transcriptomic data that is currently available for different tissues from multiple species and developmental stages (reviewed in Ref. 87), which has enabled the study of specific genes involved in a variety of functions including pigmentation, ^15^^,^^88^ vision, ^59^^,^^89^ thyroid hormone regulation of metabolism, ^79^ and sex change. ^12^^,^^76^^,^^80^^,^^82^^,^^83^

Recent analysis of gene expression patterns across tissues in *A. ocellaris* ^60^ and *A. clarkii* ^36^ revealed interesting findings pertaining to the total number of genes and unique number of genes expressed per tissue. For example, ~15% of all genes (in both species) were expressed in nearly all tissues without biased expression (as determined by the tau index which quantifies tissue-specificity ^90^) and therefore considered as housekeeping genes. On the other hand, less than 5% of all genes retrieved were absolutely specific genes (i.e., only expressed in one tissue type). Interestingly, the brain had the highest number of (total and unique) expressed genes, thus highlighting the complex role of this organ as the body control center. In *A. ocellaris*, some of the genes identified with higher expression levels in the brain are known to be involved in synapse formation in the central nervous system, chemo- and mechano-sensing of the environment, neuroplasticity, and development of spatial memory. ^60^ Whilst future research should delve deeper on the characterization of these genes, their expression levels, and the roles they may play in anemonefish phenotypic traits, both studies ^36^^,^^60^ provide the most accurate and complete transcriptomic atlas for two clownfish species to date.

A main focus of anemonefish research has been the study of the transcriptomic programs of development. Particularly, several studies have investigated developmental gene expression related to sex change. ^12^^,^^76^^,^^80^^,^^82^^,^^83^^,^^91^^–^^95^ After a first examination of the role of the aromatase gene during sex change in *A. clarkii,* ^76^ Casas *et al*. (2016) was the first transcriptome-wide study to provide insights into the genetic mechanisms governing social sex change and gonadal restructuring in clownfish. ^12^ Differential expression analyses in *A. bicinctus* revealed a complex genomic response of the brain associated with sex change that is subsequently transmitted to the gonads (see more in Section 4.1). Moreover, it identified a large number of genes, some of them well-known and others novel, that facilitated further research on sex change in anemonefish ^80^^,^^96^ as well as in other hermaphrodite fish. ^97^ The clownfish is a conspicuously colored species (possessing a bright orange body with three iridescent white bars bordered with black), and understanding the molecular basis of pigmentation has also become a fundamental question of evolutionary biology. ^2^^,^^14^^,^^16^^,^^98^ Studies on *A. ocellaris* and *A. percula* have provided insights on how pigmentation patterns are phylogenetically conserved but also exhibit developmental and environmental plasticity. ^15^^,^^88^ Other applications of transcriptomics in anemonefish research have provided in-depth characterization of visual opsins ^59^^,^^79^^,^^89^ and the rhythmic expression of internal clock genes. ^81^ More recently, Roux *et al*. (2022) provided a detailed, global overview of changes in gene expression across the post-embryonic development of the false clownfish. ^79^ Transcriptomic analysis of each of the seven developmental stages of *A. ocellaris* has revealed three distinct phases: larval development, a pivotal stage that marks the onset of metamorphosis, and metamorphosis *per se* that corresponds to the actual transformation. By identifying expression patterns of genes specifically implicated in thyroid hormone and metabolic pathways, the authors describe how the morphological and physiological changes coupled with the ecological function of *A. ocellaris* in an integrative and coherent manner. Seeing that the environment can have significant effects on metamorphosis, especially during early stages where larvae and/or juvenile phenotypes can carry-over to later life stages, this study lays the foundation for further research investigating the adaptive potential of anemonefish to climate change.

Finally, research has also examined changes in gene expression under different environmental stressors. ^21^^,^^84^^–^^86^ Experiments using qPCR analyses have revealed the differential expression of genes strongly correlated to oxidative stress resulting from UV radiation ^85^ and exposure to bisphenol A (BPA). ^86^ This last study proposed the use of cytochrome P450 1A gene ( *cyp1a*) as a biomarker for monitoring BPA pollution. Further, and in line with previous research investigating the impacts of elevated pCO _2_ on the brain transcriptome of coral reef fishes, ^99^^–^^101^ Schunter *et al*. (2021) identified changes in the expression of genes related to circadian rhythm regulators and hormone pathways in *A. percula* subjected to diel pCO _2_ fluctuations. ^21^ Notably, the authors suggest that environmental pCO _2_ fluctuations might enable reef fishes to phase-shift their clocks and adjust more successfully to ocean acidification conditions by “anticipating” pCO _2_ changes. Lastly, a new study has examined the molecular responses of *A. ocellaris* to the UV filter benzophenone-3 (BP-3) and found profound changes in the regulation of lipid metabolism that result in lipid accumulation in the liver of fish exposed to this long-time sunscreen ingredient. ^84^ As the importance of anemonefish in developmental, evolutionary, and ecological research continues to grow, but also the availability of transcriptomic resources, it is only fair to assume that so will the number of studies investigating the impacts that environmental change has on this iconic group of fish. Integrating gene expression data with physiological and other molecular measurements will certainly provide key information on adaptive phenotypes.

### 2.4 The rise of proteomics

To date, most research investigating physiological and behavioral changes of coral reef fish under warming and ocean acidification conditions has focused on transcriptomic and epigenetic modifications. ^99^^,^^101^^–^^107^ Transcriptomic expression alone, however, is not sufficient to reflect protein levels and to therefore explain genotype-phenotype relationships. ^108^ Measuring the presence and abundance of proteins is thus indispensable for the complete understanding of biological processes and cellular phenotypes, especially since post-translational modifications inferred from proteomics have been shown to be more strongly correlated to phenotypic observations than those from transcriptomics ^108^^–^^110^ ( Figure 1). Yet, technologies for quantifying the proteome are still lagging behind other “omics” fields. ^6^ Until recently, only a couple of studies have measured changes in protein expression of a closely related species to anemonefish, exposed to elevated CO _2._^6^^,^^99^^,^^111^ Proteomics can then be a powerful tool for identifying specific proteins and pathways that are crucial to stress responses, but more general for studying the evolution, biodiversity, and physiological adaptations of fish living across different environments. ^112^

Conventional methods in proteomics first focused on isolating specific proteins to study their structure and function, ^109^ which led to a very small number of intensely studied proteins over the past decades. Though protein biomarkers have facilitated a deeper understanding of various aspects of fish, ^112^^–^^115^ the use of protein-based analyses changed when new technological advancements made it possible to accurately and reliably quantify amino acids at a proteome-wide scale. ^116^^,^^117^ Indeed, mass spectrometry has become a mainstream analytical tool for proteomic profiling with diverse ecological applications. ^109^ Proteomic techniques have been classified into two categories: shotgun and targeted. Shotgun is the optimal method for discovering more proteins despite its drawback of reduced quantitative accuracy and reproducibility. On the other hand, targeted techniques are better for reproducibility when the proteins in question are known but are limited in the number of measurements and therefore the number of peptides that can be identified. ^110^^,^^118^ Particularly, iTraq (isobaric tags for relative and absolute quantification) labeled shotgun proteomics has become popular due to its use in non-model organisms. With this method, samples are labeled and processed together thus enabling the relative comparison of protein accumulation. A big limitation to this approach is, however, that the number of samples that can be compared directly is limited to a maximum of eight. Hence, pooling biological samples within one label is commonly done to increase the number of individuals that can be analyzed but results cannot be compared across experiments. ^119^ Nonetheless, previous studies measuring protein responses with the iTraq method have done so on pooled samples and found distinct proteomic patterns in fish exposed to elevated CO _2._^99^^,^^111^

A newer method that combines the advantages of both shotgun and targeted proteomics is SWATH (sequential window acquisition of all theoretical spectra)-MS, a label-free strategy capable of quantifying thousands of proteins in a single measurement. ^116^^,^^117^^,^^120^ The data are acquired on a fast, high-resolution mass spectrometer by cycling through sequential isolation windows over the entire chromatographic elution range. ^116^^,^^120^ Since it is label-free, it is relatively cheap, and it has also been shown to have high reproducibility across different labs. ^117^^,^^121^ SWATH-MS is versatile and has been used for the quantifying proteins in a number of model organisms, diseases states, and bacteria, but also characterizing different post-translational modifications (reviewed in Refs. 121, 122). A recent study laid the groundwork for using this approach on a non-model fish species. ^6^ It evaluated the performance of SWATH-MS in detecting significant proteomic expression differences in a complex experimental design of fish exposed to multiple climate change stressors. Most of all, the authors provided a guide on the efficiency, cost-effectiveness and applicability of this method in creating future proteomics references in non-model organisms aiming to identify genome-wide and ecologically relevant differential protein expression. ^6^

Certainly, the advancement of new techniques allows for the broad application of proteomics to study many aspects of anemonefishes. A few studies have investigated the proteomic responses of the spiny chromis damselfish *Acanthochromis polyacanthus* to ocean acidification, ^6^^,^^99^^,^^111^ and one provided the proteomic profile of a sea anemone species from temperate seas. ^123^ Seeing the potential of proteomics to identify ecologically relevant molecules and mechanisms, more studies should then focus on the application of these techniques to provide new insights that we have not yet obtained from genomic and/or transcriptomic expression alone. Proteomic data could unravel the processes driving symbiosis with the host anemones, sex change, complex social behaviors, and responses of anemonefish to environmental change, for example.

### 2.5 Other resources to study anemonefish biology

The combination of “omics” technologies can provide without question a wider vision of the organism of study and indicate the direction of future research. The ultimate goal of modern systems biology approaches is to integrate data from various levels of information, from gene regulatory networks, RNA and protein measurements, metabolites and cell-cell interactions, to individuals, populations and ecologies. ^124^ *In situ* hybridization, for example, is a powerful approach for studying the temporal and spatial patterns of specific genes especially because not only it enables maximum use of tissue that is difficult to obtain but can be frozen for future use. ^125^ Though protocols were originally established in zebrafish, *in situ* hybridization has also been successfully performed in anemonefish embryos ^126^ and different tissues. ^15^^,^^76^^,^^80^^,^^82^^,^^127^ Importantly, this technique revealed unique aspects of the embryogenesis of the tomato clownfish *A. frenatus* that suggest an evolutionary adaptability of the teleost developmental program. ^126^ Similarly, fluorescent *in situ* hybridization (FISH) has provided a detailed understanding of the visual system of various anemonefish species by visualizing and quantifying patterns in opsin gene expression. ^59^^,^^89^

Commercial enzyme immunoassay (EIA) kits have also proven to be a useful method to detect and quantify specific molecules. It is fast, simple, and cost-effective, and it has already been validated for measuring hormone concentrations in several species of anemonefish. ^79^^,^^128^^,^^129^ Particularly, the measurement of thyroid hormones in *A. ocellaris* has revealed an important link with metabolic regulation, morphological transformation, and behavioral changes during the transition of pelagic larvae and benthic reef associated juveniles. ^79^ Functional studies are possible now due to the development of a “low-volume” rearing protocol for anemonefish larvae, which allows the use of pharmacological approaches to alter specific biological pathways. ^49^ Experiments testing different drugs have been conducted to investigate the metamorphosis and pigmentation changes through larval development of *A. ocellaris.* ^15^^,^^79^ Cell lines have assumed an importance in molecular studies as well, especially for genetic manipulation. ^130^ Yet, so far there is only one report on cell culture from anemonefish explants. ^131^ Patkaew and colleagues (2014) described a simple and reliable method to for culturing *A. ocellaris* cells using vertebrae explants. Cytogenetic studies have further contributed to different fields of fish biology by providing basic information on the number, size and morphology of chromosomes. ^132^ Karyological analyses have been done for several anemonefish species ^133^^–^^136^ and they have consistently revealed 24 chromosomes.

As of today, no quantitative trait locus (QTL) analysis or genome-wide association studies (GWAS) have been performed in anemonefishes, thus limiting the potential for forward genetic studies (reviewed in Ref. 87). However, following a transcriptomic analysis of the mechanisms involved in sex differentiation in the Red Sea clownfish, *A. bicinctus,* ^12^ the same authors published a high-density genetic map for this species. ^13^ Essentially, this map provides a platform to study the main gene regulatory networks governing social sex change in anemonefish and other protandrous fish as well. Finally, a gene-editing protocol for applying the CRISPR/Cas9 system was recently developed in *A. ocellaris* ( Figure 1). Micro-injection of eggs was used to demonstrate the successful use of this approach at two separate target sites with 75–100% efficiency in producing biallelic F0 mutants. ^51^ Specifically, CRISPR/Cas9 knockout of the tyrosinase encoding gene ( *tyr*) involved in melanin production resulted in embryos exhibiting varying degrees of hypomelanism, thus clearly showing a loss-of-function. ^51^ This is undoubtedly a steppingstone for reverse genetic studies with exciting prospects to study the genetic basis of various unique traits of anemonefishes.

## 3. Elucidating the genomic basis of adaptation

### 3.1 Insights into the adaptive radiation of anemonefish

Anemonefish are an extraordinary example of adaptive radiation, a process driven, in this case, by the mutualistic relationship they maintain with giant sea anemones of the superfamily Actinioidea. ^137^ Notably, host sea anemones originated in the Coral Triangle region, followed by an independent geographical radiation in the Western Indian Ocean. ^138^ Thus, distribution and abundance of anemonefish are intrinsically linked to the presence and abundance of giant sea anemones. ^137^^,^^139^ Anemonefish-host anemones are, in turn, exclusively found in the tropical Indo-Pacific Ocean, with no presence in the Eastern Pacific nor Caribbean regions. ^27^^,^^33^ Anemonefish have occupied different ecological niches according to the habitat preference (e.g., reef zonation, substrate, depth) of their host sea anemones. Coexistence of multiple anemonefish species is in fact possible because of difference in host and habitat utilization. ^35^ The effect of mutualism on clownfish diversification was first examined using different nuclear and mitochondrial gene regions (e.g., 12S, 16S, ATP6-8, COI, cytochrome b, ND3, BMP-4, RAG1, RAG2), ^8^^,^^9^^,^^137^^,^^140^^,^^141^ but now the availability of high-quality genomes ^4^^,^^10^^,^^36^^,^^57^^,^^58^^,^^60^ has further clarified the phylogenetic relationships between anemonefish.

While most phylogenetic inferences ^8^^,^^9^^,^^137^^,^^140^^,^^141^ agree in the overall placement of six major species groups (the ocellaris/percula clade, the Australian clade, the skunk anemonefishes (known as the akallopisos group), the “ephippium” complex, the polymnus group, the clarkii, and the Indian clade), some discordance has been observed between mitochondrial and nuclear trees ^8^ with the main difference being the positioning of the maroon clownfish *Premnas biaculeatus* (reviewed in Refs. 24, 142). It has been long accepted to place *P. biaculeatus* together with the ocellaris/percula clade and separately from the rest of anemonefish ( Figure 2a), ^8^^,^^10^^,^^137^^–^^140^ but recent analysis ^24^^,^^60^^,^^143^ and a thorough systematic analysis of 322 damselfish species ^25^ have suggested that *Premnas* is, in fact, related to *A. ocellaris* and *A. percula* and should not be separated from the genus *Amphiprion.* The latter study reported a level of divergence within the range of what is observed between *Amphiprion* species, ^25^ which was further reinforced by Salamin and colleagues (2022), who found that gene trees estimated from 100 kb windows display an ambiguous placement for *Premnas* (either as a sister species to the ocellaris/percula clade or at the base of the tree). ^24^

Certainly, establishing a well-resolved phylogeny is critically important to understanding the evolution and genomic underpinning of anemonefish lifestyle. Nonetheless, despite the topological inconsistencies mentioned above, these studies have provided impressive insights into the adaptive radiation of clownfish. Litios *et al*. (2012) were the first to show higher rates of speciation and diversification for clownfish compared to their closest relatives without anemone mutualistic associations. Similarly, their findings also revealed a strong link between the appearance of mutualism and increased morphological evolution. ^7^ Following this study, the same authors inferred the effect of the geographical range of species on the diversification of clownfish by implementing geographic state speciation and extinction models on phylogenetic reconstructions. ^9^ Results of this study showed that most species originated in the Indo-Malay Archipelago, with one independent radiation event along the eastern coast of Africa (including the Red Sea, Maldives, and central Indian Ocean) that gave rise to seven species that now span the whole range of possible associations with sea anemones. This is interesting as instances of replicated ecological speciation over large geographic scales (of the marine realm) are quite rare, most examples being found on islands or lakes. ^144^^–^^146^

Whilst ecological speciation is likely to be the main driver of clownfish diversification, it is not the sole factor. A study by Tim and colleagues (2008) showed clear geographical subdivisions (up to ~19% of sequence divergence) in *A. percula* from Papua New Guinea and the Solomon Islands, thus suggesting that novel species might be arising by parapatric means in a region where partial isolation between subregions reinforces isolation along genetic and ecological gradients. ^147^ Hybridization has also played an important role in the evolution of anemonefish ^8^^,^^11^^,^^42^ and the several known hybrid species ^38^^–^^41^ show that it is an ongoing process. Increased glaciations and low sea levels during the Pleistocene likely promoted hybridization of many coral reef associated fish species, ^38^^,^^147^ and in the case of anemonefish this process may be further facilitated by the presence of co-existing, closely related species within the same sea anemone hosts. ^34^^,^^148^ Unlike other fish, in which it is not known what the parent species are, how often they come into contact or whether the resulting hybrids interbreed with one or both parent species, anemonefish provide a unique opportunity to understand how patterns of hybridization and introgression can be controlled by resource use and reproductive behaviour (reviewed in Ref. 38). Parent species have specific habitat requirements and may only interbreed where they overlap and co-occur. For example, the skunk clownfish *A. sandaracinos* and orange-fin anemonefish *A. chrysopterus*, which distribution overlaps in the northwestern regions of Papua New Guinea and the Solomon Islands and can co-habit in *H. crispa* and *S. mertensii* anemones, have been described as the putative parent species of the natural hybrid *A. leucokranos* ^38^ ( Figure 2c). Several other *Amphiprion* species appear to be hybridizing as well, such as *A. bicinctus* and *A. omanensis* in Socotra Island ^149^ and the historical hybridization that occurred between *A. mccullochi* and *A. akindynos* in southern Australia. ^41^ Notably, the species *A. thiellei* (probably resulting from *A. sandaracinos* and *A. ocellaris*) ^3^^,^^150^ is still under debate as there is no definitive genomic proof of its hybrid condition. ^22^ Previous studies were based on limited genetic data and did not include all species, but as progress is made in this field and availability of genomic data increases, new species may be described in the years to come.

Indeed, new whole-genomic data for all 28 species has confirmed multiple past hybridization events throughout the evolutionary history of anemonefishes. ^42^ The findings of Schmid and colleagues (2022) also shed light on the functional role of introgressive hybridization during clownfish adaptive radiation. Specifically, they show distinct phylogenetic and introgression patterns in chromosome 18 compared to the rest of the genome. This is interesting as it potentially indicates that the introgression signal was removed from the rest of the genome by extensive backcrossing but persisted on chromosome 18. This persistence might be attributed to genomic inversions that disrupt recombination and create clusters of loci controlling ecologically important traits that may consequently be fixed by natural selection or through genetic drift. ^42^^,^^142^ For example, the authors noted that genes in this chromosome are associated with the nervous system and embryonic development, and DNA damage and external stressors responses, which they suggest could be linked to advantageous traits involved in local adaptation and pre-/postzygotic isolation. ^42^ Further genomic studies are needed, however, to better characterize the various chromosomal rearrangements and the role they played in the evolution and diversification of anemonefish.

Similar to the cichlid fishes, which are famous for their large, diverse, and replicated adaptive radiations in the Great Lakes of East Africa, ^145^ a recent study found that anemonefish genomes also show major bursts of transposable elements (TE) and accelerating coding evolution. ^11^ Given that a large fraction (20–25%) of clownfish genomes consist of TE, ^4^^,^^11^^,^^36^^,^^60^ and that transposition bursts are common in teleost fishes, ^151^^,^^152^ these findings are to be expected. TE have been proposed to contribute to adaptation, speciation, and diversification processes, ^151^^,^^152^ and they may also be associated with interspecific hybridization. ^153^ Marcionetti and Salamin (2022) thus suggest that the high percentage of TE in clownfish genomes originated from two bursts of transpositions, which in turn might have played a key role in the adaptive radiation of anemonefishes. ^11^ The authors also detected increased evolutionary rates and positively selected genes (~5% of the genome) including genes with functions linked to clownfish social behavior and ecology. Surprisingly, this study did not find an excess of gene duplications in anemonefish, a remarkable finding as gene duplication has been shown to be critical for genome evolution and adaptive radiation of fish like the African cichlids. ^145^^,^^151^ Furthermore, Marcionetti and Salamin (2022) go a step further by examining the evolutionary rates and selective pressures of genes involved in the ecological divergence of clownfishes (i.e., specialist and generalist species). Altogether, the results of this study are extraordinary as they lay the foundation to understand the genomic substrate of anemonefish adaptive radiation but also open an avenue for future research investigating the genomic mechanisms governing species diversification.

### 3.2 Molecular basis of the clownfish and sea anemone mutualism

**Figure 4.**
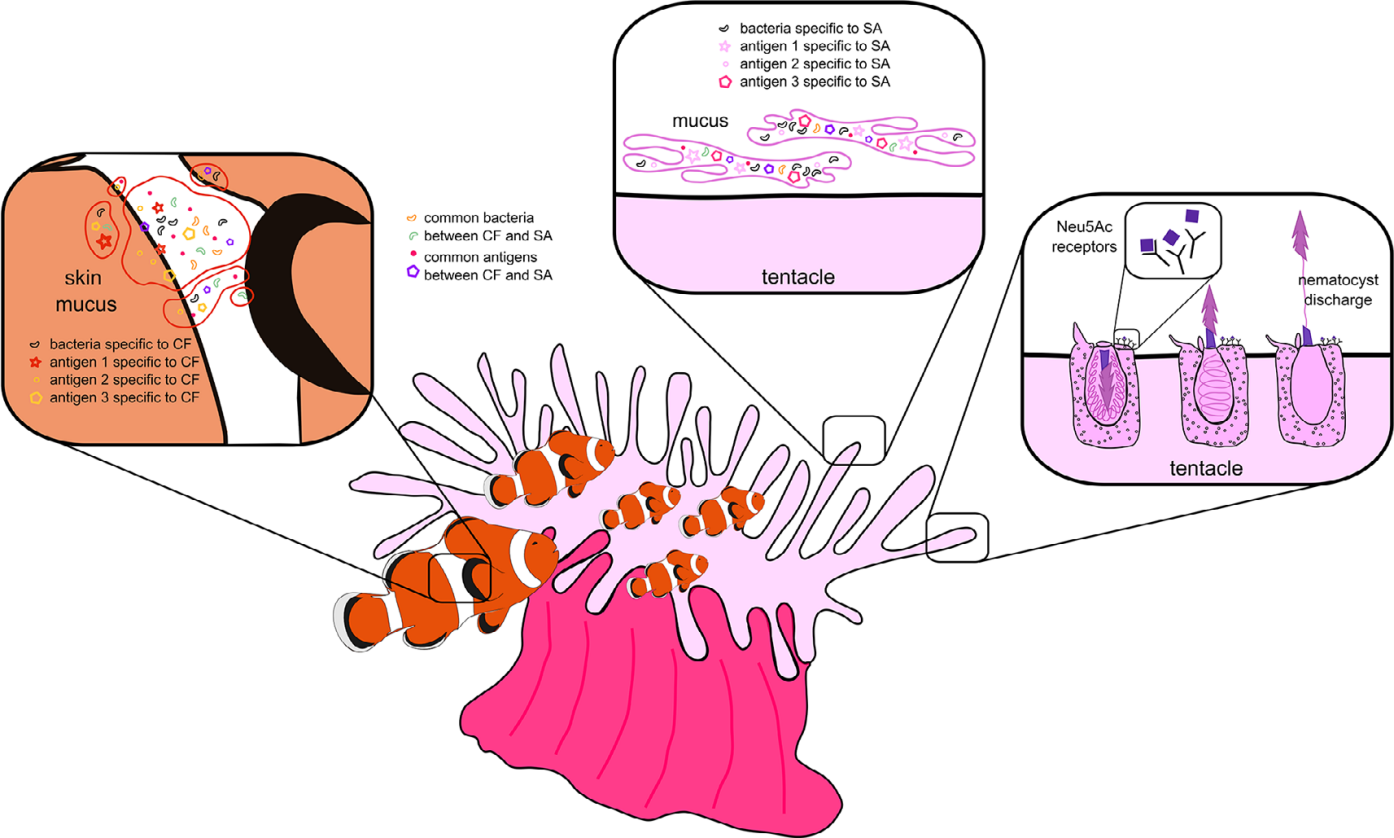
The mutualistic relationship between clownfish (CF) and sea anemones (SA) has been a long-standing question in anemonefish research. Two conflicting hypotheses have been proposed to explain how anemonefish are able to live safely in their host: 1) CF acquire antigens of the SA mucus that protects them from being stung, and 2) CF produce their own protective mucus, which either prevents nematocyst discharge by the host or protects the fish from the consequences of the sting. Particularly, N-acetylneuraminic acid (Neu5Ac) might have a critical role in the chemical recognition of the host.

Clownfish and sea anemones are perhaps one of nature’s most iconic duos. This mutualistic relationship has long fascinated biologists (first observations date back to 1868 ^154^) and it has become the subject of more recent studies investigating the evolutionary history of anemonefishes (reviewed in Section 3.1). Two aspects of this association make it particularly interesting to study: first, anemonefish can inhabit sea anemones without being harmed (unlike other fish that can be killed), and second, there is a complex species-specificity of this symbiotic relationship between the 28 species of clownfish and the 10 possible sea anemone hosts (probably related to the toxicity levels of the anemone). ^2^ The mechanism(s) underlying this mutualism remains poorly understood, but two conflicting hypotheses have been proposed to explain how anemonefish are able to live safely in their host (reviewed in Refs. 155, 156). One hypothesis proposes that anemonefish acquire certain components (e.g., antigens) of the anemone mucus that protect them from being stung (i.e., chemical camouflage). ^157^^,^^158^ Indeed, an early study ^159^ found that the mucus coating of the fish changes during the behavioral process of acclimation to resemble that of the anemone. The other hypothesis suggests that clownfish produce their own protective mucus, ^160^^–^^162^ which either prevents nematocyst discharge by the host ^163^^,^^164^ or protects the fish from the consequences of the sting. ^165^^,^^166^ Particularly, N-acetylneuraminic acid (Neu5Ac), a member of the sialic acids family, has been recognized to have a critical role in the chemical recognition of the host. ^160^^,^^164^ Indeed, it has been shown that clownfish mucus lacks this sialic acid (e.g., 1.6 mg/mL in *A. ocellaris* compared to 50.4 and 71.89 mg/mL in other reef fish species), making them “invisible” to the anemone and thus avoiding being stung. ^164^ Altogether, the above certainly suggests that the mucus of both the fish and host anemone is key for the success of this association ( Figure 4). Studies investigating the anemonefish mutualistic relationship have brought insights into the biochemical mechanisms developed by clownfish to avoid being stung by the sea anemone nematocysts ^158^^,^^159^^,^^163^^,^^164^^,^^166^ as well as the variable host specificity displayed by different *Amphiprion* species and developmental stages. ^162^^,^^167^^–^^169^ More recently, studies are leveraging the power of next-generation sequencing technologies to better understand the genetic basis ^10^ and potential microbial role in clownfish adaptation to sea anemones. ^17^^–^^19^^,^^170^

Only recently, candidate genes that may grant anemonefish protection from the host toxins were identified for the first time. ^10^ Marcionetti and colleagues (2019) highlighted the genes *versican core protein* and *protein O-GlcNAse* as particularly interesting due to their functional link with N-acetylated sugars. ^10^ *Versican core protein* is known to be a critical extracellular matrix regulator of immunity and inflammation ^171^ that interacts with several matrix molecules including glycosaminoglycans such as N-acetylglucosamine (GlcNAc). ^172^ Expression of *versican core protein* in clownfish skin is thought to bind to N-acetylated sugars, masking their detection by the host chemoreceptors and therefore preventing nematocyst discharge (reviewed in Ref. 10). On the other hand, *protein O-GlcNAse* has the potential to cleave N-acetylated sugars from different cell surface molecules ^173^ and has also been found to be expressed in anemonefish epidermis. ^10^ Additionally, clownfish-specific duplicated genes involved in immunity (e.g., *T cell receptor alpha*) and detoxification (e.g., *cytochrome P450*, *glutathione S-transferasas*) responses were also identified. ^10^ Conclusions regarding the potential role of these genes in the protection from anemone secreted toxins cannot be drawn, however, without further experimental evidence.

Given that the skin is the first line of physical contact between clownfish and sea anemones, and that epithelial microbial communities are important drivers of symbiotic interactions, ^174^ more studies are now investigating the microbial signatures of the clownfish-sea anemone mutualism. ^17^^–^^19^^,^^170^ Pratte *et al*. (2018) were the first to show that contact with host anemones can significantly reshape the clownfish skin microbiome. ^17^ Their findings revealed a drastic shift in the epithelial bacterial communities of *A. clarkii* within one week of association with their host. Interestingly, they also showed that these changes are reversible. Following this study, Roux and colleagues (2019) examined the microbiome of *A. ocellaris* and *H. magnifica* mucus simultaneously before and after initiation of the symbiotic relationship. ^18^ The authors found distinct microbial signatures for each symbiont before initial contact (e.g., Alteromonadaceae dominated the clownfish skin bacterial taxa whereas sea anemone mucus was mainly composed of Pseudoalteromonadaceae and Endozoicomonadaceae) that were subsequently modified during the establishment of symbiosis (e.g., fish-anemone microbiota shared the families Haliangaceae, Pseudoalteromonadaceae, and Saprospiraceae). Until then, this was the only study to have tested the effect of clownfish association in the mucosal microbiota of the sea anemone and shown microbial convergence between both partners (but see Refs. 19, 170). Notably, the latter seems to substantiate the hypothesis that anemonefish cover themselves with their host mucus to avoid being stung. ^157^^,^^158^

A more recent study examined shifts on the skin microbiota in clownfish when in direct contact with its host (i.e., fish and anemone are in the same tank) but also tested the effect of “remote interaction” (i.e., fish and anemone are in separate tanks, both connected to the same water flow) on the epithelial microbiome restructuration in both partners. ^19^ The results of this study are compelling as they provide evidence of a strong water-mediated chemical communication between both symbiotic partners (as seen by the gradual convergence in the microbial communities of the fish and its host when they are both placed in the same water system). Interestingly, increasing abundances of three sequence variants closely related to a tyrosinase-producing *Cellulophaga tyrosinoxydans* bacterium were observed during microbiota convergence. Noteworthy, bacterial tyrosinases (which catalyze melanin synthesis) have been shown to be immunologically active compounds, providing skin protection against radiation, viral agents, immunogens, and/or toxic compounds. ^88^^,^^175^ Whether convergence of microbial communities might play any role in the symbiotic relationship between clownfish and sea anemones remains to be determined nonetheless. Metagenomic and metatranscriptomic approaches would be the next step to gain a deeper understanding on specific gene functions and expression patterns of the microbial communities involved in this mutualism. Finally, it is worth mentioning a study by Titus *et al*. (2020) as it shows for the first time the effect of host identity and symbiotic association on the functional diversity and composition of the microbiome. ^170^ Microbiota of different anemones ( *C. adhaesivum*, *E. quadricolor*, *H. aurora*, *H. magnifica*, and *S. mertensii*) harboring the same species of clownfish ( *A. nigripes* or *A. clarkii*) were more similar to each other than to that of anemones that were hosts to different species of anemonefish. Furthermore, this is the only study examining *in situ* microbiomes so far. Experiments in field conditions are needed to ultimately establish the role of microbial communities in the clownfish-sea anemone symbioses.

### 3.3 Phenotypic plasticity and genetic assimilation in development and evolution of anemonefish

Adaptation to changing environments has long been a central question in evolution. ^176^ Phenotypic plasticity, the ability of a species to produce multiple phenotypes (e.g., alternative morphology, physiological state, behavioral response) from a single genotype, has been shown in many terrestrial and aquatic organisms and is critically important for adaptation of populations to local environments. Environmentally induced non-genetic effects on phenotypes can alter the strength and direction of selection affecting transmitted gene frequencies by shifting the range of phenotypes expressed. In such cases, a phenotype, which initially is produced only in response to a specific environment, becomes assimilated genetically so that it is formed even in the absence of the environmental influence that had been necessary before (reviewed in Refs. 176, 177). In anemonefish, color polymorphisms within populations have received considerable attention and have been attributed to developmental plasticity (reviewed in Ref. 178). The latter referring to the ubiquitous ability to adjust phenotypic development in response to environmental cues experienced in early life stages. ^179^

**Figure 5.**
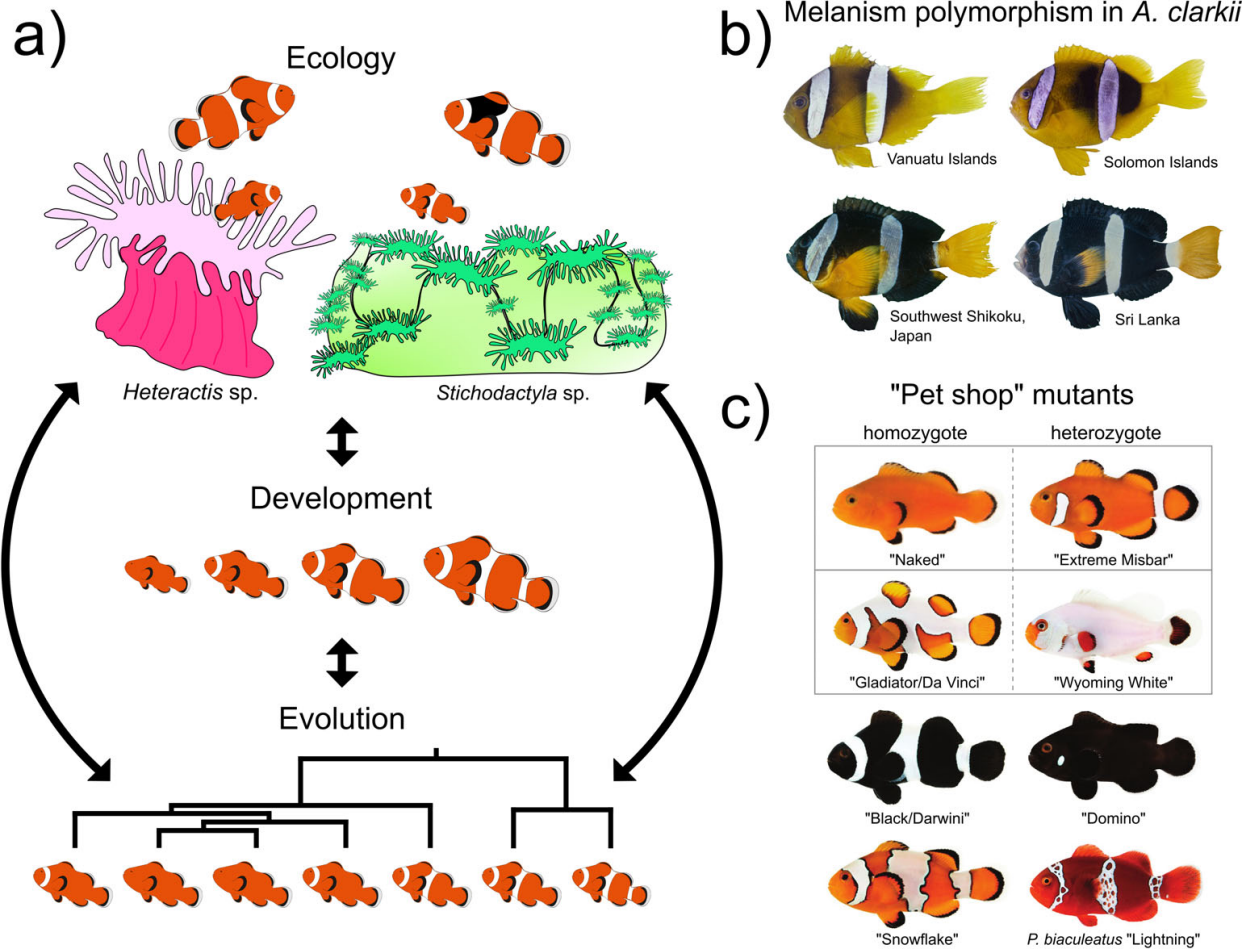
New insights into the processes generating complex pigmentation patterns in reef fish. a) Color patterns in anemonefish can vary greatly depending on their ecology, development, and evolution (adapted from Refs. 15, 178). White bars could be necessary for species recognition and could be adaptive for camouflage or even used as an aposematic signal. Pigmentation polymorphisms have also been observed in *Amphiprion percula* living in *Heteractis* or *Stichodactyla* anemones: 1) juveniles in *Heteractis* exhibit a delayed white bar formation and 2) adults in *Stichodactyla* show higher melanism. The three white bars arise sequentially from anterior to posterior body parts during ontogenesis whereas during evolution, bars are lost in the opposite sequence of ontogenesis (from the posterior to anterior region). b) Natural melanism polymorphism observed in *Amphiprion clarkii* (adapted from Ref. 36). c) Examples of *Amphiprion ocellaris* color mutants available from aquaculture companies: naked phenotypes (“Naked” and “Extreme Misbar”), phenotypes with extra white markings (“Gladiator/Da Vinci”) to an almost nearly complete white colored body (“Wyoming White”), melanic phenotypes (“Black/Darwin” and “Domino”), and phenotypes with irregular patterning (“Snowflake” and the *Premnas biaculeatus* “Lightning”) (adapted from Ref. 16).

Phenotypic (developmental) plasticity as a phenomenon enables the study of the link between gene expression and phenotype since it involves the production of various phenotypes without genetic changes. Species with adult individuals that can be experimentally induced to transition between distinct phenotypes are highly valuable. They make it possible to isolate phenotypic effects of gene expression by comparing the gene expression profiles of groups of individuals who differ in their phenotypes due to plasticity rather than genetic differences. ^180^ Such is the case of the yellowtail clownfish *A. clarkii*, for example, a species known for showing a high degree of melanism polymorphism. ^72^ Particularly, melanism in *A. clarkii* varies with social rank, ^37^^,^^181^ local variations in habitat (e.g., temperature), ^182^ and host anemone. ^72^ Other species of anemonefish ( *A. chrysopterus*, *A. percula*, *A. polymnus*) also exhibit polymorphic melanistic morphs depending on the host they associate with. Indeed, observations have noted that fish inhabiting *Stichodactyla* spp. are generally darker (i.e., more melanic), whereas individuals in other anemones (e.g., *Heteractis* spp., *Entacmaea quadricolor*) tend to be more orange. ^72^ Moreover, Salis *et al*. (2021) recently showed that the developmental timing of white bar formation in juvenile *A. percula* depends on the anemone species to which they have recruited. ^20^ Specifically, earlier formation of white bars when clownfish developed in *Stichodactyla gigantea* rather than *Heteractis magnifica* was observed ( Figure 5a). Using a combined approach of transcriptomic analysis and pharmacological treatments, the authors showed that thyroid hormones are essential in modulating the timing of adult color pattern formation and which are, in turn, associated with ecological differences. This study offers great promises to understand the genomic and developmental basis of plastic phenotypes observed in wild clownfish.

## 4. Genomic architecture underlying anemonefish phenotypes

### 4.1 Sex change

Fish exhibit extraordinary sexual plasticity, changing sex naturally as part of their life cycle ^12^^,^^183^ or because of environmental stressors. ^184^^,^^185^ Indeed, sequential hermaphroditism has been reported for at least 27 teleost families spread across nine orders in three different forms: protogynous (i.e., female to male), protandrous (i.e., male to female), or bidirectional sex change (reviewed in Ref. 186). From these, protandry is rarer among teleosts, occurring sporadically across six families including anemonefish. ^186^ Anemonefish are particularly interesting, as contrary to most protandrous species, which need to attain a threshold age/size to change sex, their sex change is regulated socially. ^96^ The first molecular characterization of sex change in anemonefish dates to the late 2000s when Miura and colleagues (2008) performed immunohistochemical detection to examine the expression of the aromatase gene ( *cyp19a1*) during gonadal development of *A. clarkii.* ^187^ Soon after, numerous studies started investigating the expression patterns of different hormones involved in sex change by using qPCR. ^76^^,^^82^^,^^83^^,^^91^^–^^95^ However, it was not until the year 2016 that Casas *et al*. explored the transcriptome-wide expression landscape during sex change in a clownfish. ^12^ To this, followed a high-density map containing tens of genes involved in sex differentiation. ^13^ Collectively, these studies identified several genes (and their location) that may be important orchestrators of sex change and gonadal transformation in anemonefish. Importantly, these studies are also shedding new light on the gene regulatory mechanisms underlying functional hermaphroditism in fish. ^96^^,^^97^^,^^186^

Transcriptomic analysis of the Red Sea clownfish *A. bicinctus* showed a gradual decline in male-related gene expression and up-regulation of female-pathway genes as the gonads transitioned from ovotestis to ovaries (reviewed in Refs. 12, 96, 97). Active feminization of the (male) brain starts two weeks after the female is removed (i.e., social cue) so that the transcriptional response is subsequently transmitted to the gonads where differential expression and histological changes can be clearly observed after three to four weeks. Specifically, *cyp19a1* (steroidogenic enzyme operating in the female pathway by converting androgens into estrogens) exhibited increasing expression in the brain of transitional males. This might be, at the same time, regulated by the expression of two other genes: *sox6* (transcription factor involved in spermatogenesis) and *foxp4* (transcription regulator with an important role in sexual development). A specific mechanism of action remains to be established though. Changes at the gonadal level, on the other hand, are also driven by the over-expression of *cyp19a1* which then triggers the up-regulation and down-regulation of *foxl2* (transcription factor involved in ovarian differentiation) and *dmrt1* (gene involved in the development and regression of testis), respectively. Based on this, Casas *et al*. (2016) propose a feedback loop combining transcriptional regulation with steroid hormonal activity where *dmrt1* and *foxl2* regulate the production of *cyp19a1* and thereby, gonadal restructuring during sex change in clownfish. ^12^

Importantly, the same molecular pathways have been described for another *Amphiprion* species. ^80^ Wang and colleagues (2022) also conducted comparative transcriptomic analysis of gonads of the complete social group (females, males, and non-breeders). In addition to this, they also performed *in situ* hybridization to show expression localization of *foxl2* and *dmrt1.* Consistent with previous findings, *foxl2* could only be detected in the granulosa cells of oocytes in female gonads, whereas signals of *dmrt1* were detected in spermatogonia and spermatocytes in male gonads. ^80^ New research using exogenous steroid (i.e., estradiol and cortisol) treatments combined with qPCR have further validated the role of *cyp19a1* in feminization in *A. ocellaris.* ^83^ Particularly, cortisol pellet-fed fish exhibited a decline in expression of *cyp19a1* and dominant behavior intensity. The authors showed that high cortisol concentrations inhibit transcription of the aromatase gene, which results in masculinization. Thus, suggesting that the interaction between cortisol and aromatase might play a pivotal role in sex change of anemonefish. The feminization of the brain in anemonefish is inarguably an active process, and the timing and speed at which these changes occur remain a compelling and active field of research. The process spans a wide time frame, with brain expression profiles changing relatively rapidly after female removal (0 to 11 days in males and 15 to 30 days in transitional males ^12^) and complete gonadal changes taking much longer (sometimes over the course of several years). ^188^ Altogether, anemonefish provide a unique opportunity to explore the molecular, biochemical, and physiological mechanisms underlying sex change in vertebrates.

### 4.2 Pigmentation and color patterns

Color patterns in adult fish provide a unique opportunity to study the interplay between ecology, development, and genetics that is the basis for trait diversification (reviewed in Ref. 98). Indeed, fish have the highest number of pigment cell types (chromatophores) and color diversity among vertebrates, which is not surprising considering the ample pigmentation gene repertoire they have as a result of undergoing a third (and fourth, in the case of salmonids) whole genome duplication round. ^189^^,^^190^ Body coloration of fish varies greatly, different species and/or life stages display diverse combinations of spots, stripes, bands, eyespots, etc. ^98^ that can also change depending on environmental cues and geographic distribution ^20^^,^^72^^,^^181^^,^^182^^,^^191^^–^^193^ ( Figure 5a). Certainly, color patterns have clear ecological and behavioral significance, with functions ranging from recognition of conspecifics, to avoidance of predators, sexual attraction, and protection against ultraviolet radiation. ^98^ In anemonefish, pigmentation plays a key role in the complex hierarchical social system they have. Young recruits are colored distinctly different than older juveniles to potentially avoid antagonistic and aggressive behaviors from the larger individuals. ^178^ Loss of white vertical bars during ontogeny has indeed been observed in multiple *Amphiprion* species. ^14^ Mitchell and colleagues (2022) further showed that UV reflectance in anemonefish (from their orange and white bars) has a functional role in modulating aggression and signaling submissiveness in family groups. ^194^

Salis and colleagues (2018) mapped the occurrence of these bars throughout the phylogenetic tree and showed that the diversification of color patterns in anemonefish is the result of successive (posterior to anterior) losses of bars during clownfish radiation. The sequential appearance/disappearance of white bars during the development of distantly related species is remarkable as it suggests a highly conserved mechanism of pigmentation pattern ontogeny across anemonefish ^14^ ( Figure 5a). Different phylogenetic approaches have also shown evolutionary pathways linking the number of bars with host specificity and host toxicity (i.e., fish with fewer bars associate with fewer and more toxic anemone species than fish with higher number of bars). ^195^ Color polymorphisms are also known to occur frequently in anemonefish (also see Section 3.3), whether they are rare natural variants ^43^^,^^72^ or mutants found in pet shops. ^16^ Widely distributed species such as *A. clarkii* exhibit great intraspecific polymorphism for melanic pigmentation according to geographical variation and environmental conditions ^27^^,^^72^^,^^181^^,^^182^ ( Figure 5b). Interestingly, a recent study revealed higher copy numbers of the receptor protein kinase *erbb3b* gene (which is involved in melanocyte development) in *A. clarkii* compared to other anemonefish, thus implying a possible link between *erbb3b* and the natural melanism polymorphism observed in this species. ^36^ Morphotypes such as albinism or individuals with no bands in species that usually have, are never or very rarely observed in the wild but can be found in the aquarium trade industry (reviewed in Ref. 16). In the wild, mutations that result in such drastic color pattern alterations are likely to have a negative effect on the survival of individuals and are therefore negatively selected against, but they can be bred for several generations in aquaculture. As global trade in ornamental fish has become a multi-billion dollar industry (reviewed in Ref. 196), many color mutations have been characterized. ^16^ For example, mutations related to “naked” phenotypes (i.e., absence of white bars) may be specifically caused by genes such as *ltk* (leucocyte tyrosine kinase), *sox10* (SRY-related HMG-box) and endothelin receptors *edn3b* and *ednr3b* that are responsible for iridophore specification. Klann *et al*. (2021) further review the mutations underlying many of the pigmentation variants known for anemonefish until now and present a global picture of their origins and crosses ^16^ ( Figure 5c).

Studying pigmentation also allows further understanding of the cellular basis of adult form, as the cells that produce diverse color patterns are readily visible in the skin during development. ^15^^,^^54^ Thus far, however, genetic and cellular mechanisms of lineage specification, differentiation, and morphogenesis during pigment pattern formation have been studied most extensively in zebrafish (reviewed in Ref. 197). It is only until recently that Salis *et al*. (2018, 2021) described in detail the emergence of pigmentation during embryonic development in the anemonefishes *A. ocellaris* and *A. perideraion.* ^54^^,^^198^ In another study, the same authors also investigated the molecular basis of white barring in clownfish. ^15^ Using transcriptomic approaches, they showed that white skin in clownfish have a transcriptomic signature of purine-containing iridophores, similar zebrafish and oppose to leucophores in medaka, for example. Particularly, four genes ( *fhl2a* – four and a half LIM domains 2a, *pnp4a* – purine nucleoside phosphorylase 4a, *prtfdc1* – phosphoribosyl transferase domain containing 1b, and *tfec* – transcription factor EC) were inferred to be essential for the development and function of iridophores. Great progress in identifying the genetic and cellular bases of pigment patterns formation in anemonefish is being made in our laboratory, nonetheless there is still much to understand. Beyond zebrafish, a classical fish model for vertebrate biology, we are only just beginning to understand how the molecular mechanisms underlying the diverse pigmentation in clownfish and other teleosts, but the emerging genomic and imaging technologies offer a promising future in this field.

### 4.3 Longevity and lifespan

The evolutionary theory of aging predicts that individuals with low extrinsic mortality will show delayed senescence (i.e., the process of physiological deterioration with age) and increased lifespan (reviewed in Ref. 199). It is important to note that although the terms “lifespan” and “longevity” may sound interchangeable, they hold distinct meanings when discussing life expectancy. Lifespan refers to the maximum potential duration of life for a given species or population. Longevity, on the other hand, describes the ability to live a long life beyond the species-specific average age at death (reviewed in Refs. 200, 201). To illustrate, if the average lifespan of a species is 10 years, for example, it means that, on average, individuals of that species are expected to live 10 years. However, if the longevity of the same species is 10 years, then it means that some individuals may live up to 10 years, but the average lifespan may still be much lower than 10 years. In the case of anemonefish, their slow aging and increased longevity is linked to their low extrinsic mortality, which is in turn correlated to the low predatory pressure they experience due to their association with sea anemones. ^202^ Buston and García (2007) estimated a life expectancy of 30 years for wild *A. percula,* ^202^ and similarly for *A. ocellaris* and *A. melanopus* in captivity. ^203^ Noteworthy, this estimate is two times greater than the longevity estimated for any other pomacentrid and up to six times greater than the longevity expected for a fish of that size. ^202^ Anemonefish therefore stand out as quite unique under this criterion also.

Transcriptome sequencing of several *Amphiprion* species ^203^ revealed 157 positively selected genes, several of which are related to processes linked to xenobiotic and glutathione metabolism, detoxification, mitochondrial translation, inflammation, and autophagy. In particular, the authors found a positive selection of two lysosomal membrane proteins ( *LAMP2* and *CD63*) known for playing an important role in chaperone-mediated autophagy, lysosomal protein degradation, adaptive immune response, and apoptosis. These results are consistent with earlier findings that have associated lysosomal function as one of the key hallmarks of aging, ^199^ thus implying that positive selection of lysosomal genes plays an important role in the evolution of exceptionally long life of anemonefish. ^203^ Interestingly, this study also showed evolutionary convergence with the short-lived killifish and the long-lived mole rat. Signs of convergence were observed for genes and pathways involved in the biogenesis of mitochondrially-encoded proteins with the remarkable observation that *MTERF* (mitochondrial transcription termination factor 1) is under positive selection in all three taxa. This parallels previous evidence suggesting mitochondrial biogenesis as a core genetic substrate in the evolution of lifespan. ^199^ Furthermore, the observation that the same pathway is under positive selection in both exceptionally short- and long-lived species indicates that the same genetic architecture underlies both evolution of lifespan and longevity. ^199^^,^^203^ Altogether, this makes anemonefish the first long-living fish model for aging research. ^204^

After exploring the remarkable long lifespan of anemonefish, a few other compelling questions arise: Do anemonefish have undetermined growth, growing too large for their host sea anemone and eventually having to move to another and larger anemone? Does the longevity of the host sea anemones parallel that of anemonefish? In a groundbreaking milestone, Rueger and colleagues (2022) ^205^ provided the first-ever experimental evidence for the first question. Their study sheds light on the remarkable growth plasticity of anemonefish in response to their mutualistic interaction with sea anemones, that is, anemonefish adjust their growth rate to make sure they are the ideal size for their hosts. Thus, emphasizing the crucial role of mutualisms in shaping species’ adaptations and ecological relationships. Moreover, this research opens up exciting new avenues for exploring the underlying molecular mechanisms behind this phenomenon: What are the cues necessary for the fish to decide how large it needs to be? Having explored the growth plasticity of anemonefish, we now shift the focus now to the longevity of the host sea anemone and its intricate association with the presence and size of their anemonefish counterparts. Various studies have shown that giant sea anemones are short lived compared to their fish symbionts, with some species like *E. quadricolor* and *H. crispa* having turnover times of only 3–5 years. ^206^^–^^208^ The short lifespans of these host anemones certainly affect their resident anemonefish as they need to migrate among hosts during their lifetimes, and do so when space becomes available nearby. As a result, some anemonefish may rely on the presence of multiple anemone hosts within a relatively small area of reef, leading to potential constraints on their lifespans due to host turnover. This is particularly evident in areas with low and declining host abundance. ^206^

## 5. Evolutionary genomics of complex traits in anemonefish

Adding to the many traits that make these fishes fascinating, anemonefish also exhibit interesting behaviors such as social group formation and parental care. The mechanisms involved in social evolution in clownfishes, and more specifically the interspecific variation in the genetic benefits and ecological constraints of forming social groups, have been a major focus of study in anemonefish research (reviewed in Ref. 209). Parental behaviors in anemonefish have also been well described, with a growing number of studies investigating the neural pathways and brain regions regulating parental care. Particularly, there is an interest in understanding the plasticity of parental care in response to changes in ecological context (i.e., resource availability) and social roles (across sex change) (reviewed in Ref. 210). Behavioral genomics is still in its infancy; the complexity of individual and group behaviors, and the highly polygenic nature of these traits, make it challenging to study the mechanistic links between genes and behavior. Nonetheless, with the recent advent of (affordable) “omics” approaches, it is increasingly possible to identify the precise genetic contributors of a wide diversity of behaviors, allowing for new insights into how behavior evolves in the wild. More studies are now focusing on investigating the role of genetic/genomic variation (from DNA sequences to brain gene expression, to neuronal dynamics, to gene regulatory networks) to understand the genetic/genomic bases of behavior. ^211^ The establishment of anemonefish as a model organism in different biological disciplines, and the availability of high-quality reference genomes and transcriptomic data will certainly facilitate the quest for answers on clownfish behaviors. Seeing that behavioral traits are complex, carefully designed experiments are needed to disentangle individual and group behaviors. The plastic nature of behavior and the likelihood that many genes with small effects are involved also makes quantifying the role of natural genetic variation difficult. ^212^ Newer tools have been developed to test candidate genes with behavioral functions. James and Bell (2021) used virus-mediated transgenesis (through direct brain injection) to study how overexpression of certain genes affected aggressive behaviors in the fish model *Gasterosteus aculeatus.* ^213^ Indeed, species such as the threespine stickleback and zebrafish have long been the subject of behavioral genetics, ^213^^–^^217^ providing valuable insights that can potentially be transferred to anemonefish.

## 6. Conclusions and perspectives

Anemonefish have become an invaluable model system for answering some of the most fundamental and long-standing questions in evolutionary genomics. How do speciation events occur, and what are the underlying genetic mechanisms driving this process? What genetic changes underlie the development of morphological, physiological, and behavioral traits? How do organisms adapt to their environments, and what role does natural selection play in shaping their genomic architecture? How can we integrate genomic data from multiple species to gain insights into the evolutionary processes shaping biodiversity? Future research will most likely be more integrative, incorporating not only the topics discussed here, but also other fields such as ecotoxicology and neuroendocrinology, as well as continued integration with ecology and behavior. Recent technological advancements have facilitated the generation of huge amounts of genomic, transcriptomic, and proteomic data that can be leveraged to answer complex questions pertaining to the many traits that make anemonefish extraordinary. Importantly, one of these traits is that, compared to most marine fish, anemonefish (almost) never abandon their host sea anemone thus making them ideal subjects for long-term monitoring studies. Indeed, the first multigenerational pedigree for a marine fish population was constructed using data from a 10-year genetic survey of *A. percula* from Kimbe Bay in Papua New Guinea. ^48^ Such genealogy provides a unique opportunity to study how maternal effect, environment or philopatry can shape wild fish populations, for example. Long-term genomic monitoring will certainly become a powerful tool to assess species and ecosystem vulnerability to environmental change. Anemonefishes are becoming a mainstay to study adaptive responses of marine fish to climate change and ocean acidification, a body of work that will only continue to grow. Finally, anemonefishes are now strongly positioned to exploit rapidly emerging tools such as CRISPR/Cas9 which will be crucial to gain insights into the molecular basis underlying specific phenotypes and genetic variants.

## Data Availability

No data are associated with this article.
