# Supplementary material for: Anemonefishes: A model system for evolutionary genomics
Source: F1000Res. 2023 Oct 27;12:204. Originally published 2023 Feb 21. [Version 2] doi: 10.12688/f1000research.130752.2 (PMC10624958; doi:10.12688/f1000research.130752.2)
Supplement: Peer review history

# Reviewer report: Reviewer response for version 2

**DOI:** 10.5256/f1000research.153773.r218891

**Published:** 2023-11-03

**Authors:** Kerstin Johannesson

**Affiliations:** 1 Department of Marine Sciences, Tjärnö Marine Laboratory, Goteborgs universitet, Gothenburg, Västra Götaland County, Sweden

I think the authors have done a great job and fixed the issues that I was a bit concerned about by giving more details. So I am happy with the report as it is.

Is the review written in accessible language?

Yes

Are all factual statements correct and adequately supported by citations?

Yes

Are the conclusions drawn appropriate in the context of the current research literature?

Yes

Is the topic of the review discussed comprehensively in the context of the current literature?

Yes

Reviewer Expertise:

NA

I confirm that I have read this submission and believe that I have an appropriate level of expertise to confirm that it is of an acceptable scientific standard.

# Reviewer report: Reviewer response for version 2

**DOI:** 10.5256/f1000research.153773.r218892

**Published:** 2023-10-30

**Authors:** Fabio Cortesi

**Affiliations:** 1 School of the Environment and Queensland Brain Institute, The University of Queensland, Brisbane, QLD, Australia

I thank the authors for taking the time and doing an excellent job addressing my comments. I do not have any further comments to make.

 Congratulations on a very nice review.

Is the review written in accessible language?

Yes

Are all factual statements correct and adequately supported by citations?

Yes

Are the conclusions drawn appropriate in the context of the current research literature?

Yes

Is the topic of the review discussed comprehensively in the context of the current literature?

Yes

Reviewer Expertise:

Evolution, neuroethology, marine biology, ichthyology, sensory biology, molecular ecology, behavioural ecology, genomics, transcriptomics

I confirm that I have read this submission and believe that I have an appropriate level of expertise to confirm that it is of an acceptable scientific standard.

# Reviewer report: Reviewer response for version 1

**DOI:** 10.5256/f1000research.143530.r170667

**Published:** 2023-05-18

**Authors:** Kerstin Johannesson

**Affiliations:** 1 Department of Marine Sciences, Tjärnö Marine Laboratory, Goteborgs universitet, Gothenburg, Västra Götaland County, Sweden

This review take a broad grip on the anemonefish genus, describing both basic and interesting facts on the biology of these fascinating fish, and (which is the focus on the review) how these species are now emerging as excellent models for studies of evolutionary questions in a broad sense. I congratulate the authors to a comprehensive and also very nicely illustrated. I learnt a lot about anemonefishes reading it. As I am not in the field of anemonefishes, I have some comments that the authors might consider to further clarify some minor parts of the text.

 The first issue is really trivial for an expert, but for a non-expert it is a bit confusing to use both "anemonefish" and "clownfish" interchangeably, and without pointing out that these are synonymous. It took me a while to find out. Maybe better to stick to anemonefish throughout?

 In Fig. 3c, I am confused about the red ribbons that are representing inversions and translocations. In some places it seems as if these corresponded to one whole chromosome, and so what is actually going on at chromosome 3 in A. *ocellaris* and chromosome 4 in A. *percula*. Are these just not the same chromosome that have been given different numbers in the two species? Or is there actually also inversions or translocations involved?

 Under 2.4. Last sentence - why only in extreme environments?

 Under 2.5. Second sentence - the long parenthesis makes the sentence uneasy to read

 Under 3. First paragraph. Sea anemones are found globally, and so it is not obvious to me why the anemonefish are confined only to the tropical areas of western Pacific and the Indian Ocean. For example, in NE Atlantic, there are shrimps that associate with sea anemones in much the same way as the anemonefish, and so, why can there not be other genera of fish that have evolved this habit?

 3. Five paragraph. Not sure what you mean by “consequently fixed by natural selection” - do you mean that the inversion is always fixed different (one arrangement at frequency 1.0 in one species, and the other arrangement fixed at 1.0 in the other species? One alternative would be that the inversion is polymorphic in both species, so this need to be clarified. What is here referred to as “supergenes” is also somewhat unclear. Usually, the definition of a supergene is that is e.g. an inversion (or some other low-recombination region) which remains polymorphic within populations - and so this does not match the statement of “fixed by selection”....

 Under 4.3. One obvious question after the presentation of the extremely long life-span is, does anemone fish have undetermined growth? That is, do they grow to large for their anemone, and so have to move to another and larger anemone? (Also, are the anemones also similarly long-lived?)

 Under 4.3, second paragraph. What is the difference between “lifespan” and “longevity” in the next last sentence of this paragraph?

 Under 6. First sentence - please spell out which are these fundamental questions you are referring to.

 That the fish never abandon their anemone is a very interesting fact that should perhaps be highlighted earlier (or I did miss it when reading), together with the very long lifespan, to illustrate what is the unique features of the anemonefish as a model.

Is the review written in accessible language?

Yes

Are all factual statements correct and adequately supported by citations?

Yes

Are the conclusions drawn appropriate in the context of the current research literature?

Yes

Is the topic of the review discussed comprehensively in the context of the current literature?

Yes

Reviewer Expertise:

Molecular ecology, evolutionary genetics, population genetics, hybrid zones and speciation

I confirm that I have read this submission and believe that I have an appropriate level of expertise to confirm that it is of an acceptable scientific standard, however I have significant reservations, as outlined above.

# Response

**Published:** 2023-08-03

**Authors:** Marcela Herrera Sarrias

**Affiliations:** Okinawa Institute of Science and Technology, Japan

The first issue is really trivial for an expert, but for a non-expert it is a bit confusing to use both "anemonefish" and "clownfish" interchangeably, and without pointing out that these are synonymous. It took me a while to find out. Maybe better to stick to anemonefish throughout?

**The reviewer is right, and this has been clarified. The first paragraph of section #2 now reads: “** **Before presenting the various contributions made in the field of anemonefish research, we feel it is essential to clarify the difference between the terms "anemonefish” and “clownfish” both of which are used throughout this review. Conventionally, the English name “anemonefish” has been associated with the distinctive symbiosis between these fish species and giant sea anemones, while the term “clownfish” highlights their vibrant colors and bold behavior. In line with the prevailing practice among researchers in this field, we have opted to refer to them as “anemonefish” to acknowledge the pivotal importance of their symbiotic relationship with giant sea anemones, a key aspect that profoundly influences their biology. However, for *Amphiprion ocellaris* and *Amphiprion percula*, two closely related species forming a natural subgroup within anemonefish, we do use the term “clownfish”.”**

 In Fig. 3c, I am confused about the red ribbons that are representing inversions and translocations. In some places it seems as if these corresponded to one whole chromosome, and so what is actually going on at chromosome 3 in A. *ocellaris* and chromosome 4 in A. *percula*. Are these just not the same chromosome that have been given different numbers in the two species? Or is there actually also inversions or translocations involved?

**As indicated in the legend of this figure, the red ribbons depicted represent various chromosomal rearrangements, including translocations and inversions. Chromosomes in both species have been designated based on their size following an orderly arrangement from the largest to the smallest. This has been further clarified in the legend of the figure.**

 Under 2.4. Last sentence - why only in extreme environments?

**This has been modified so it now reads: “** **Proteomics can then be a powerful tool for identifying specific proteins and pathways that are crucial to stress responses, but more general for studying the evolution, biodiversity, and physiological adaptations of fish living** **across different** **environments.** **”**

 Under 2.5. Second sentence - the long parenthesis makes the sentence uneasy to read

**As suggested by the reviewer, this has been modified so it now reads: “** **The ultimate goal of modern systems biology approaches is to integrate** **data from** **various** **levels of information** **,** **from gene regulatory networks, RNA and protein measurements, metabolites and cell-cell interactions, to individuals, populations and ecologies.** **”**

 Under 3. First paragraph. Sea anemones are found globally, and so it is not obvious to me why the anemonefish are confined only to the tropical areas of western Pacific and the Indian Ocean. For example, in NE Atlantic, there are shrimps that associate with sea anemones in much the same way as the anemonefish, and so, why can there not be other genera of fish that have evolved this habit?

**The reviewer is right, and we apologize for the lack of clarity. We have modified this so it now reads: “** **Anemonefish are an extraordinary example of adaptive radiation, a process driven, in this case, by the mutualistic relationship they maintain with** **giant** **sea anemones** **of the superfamily Actinioidea** **.** **^135^** **Notably, host sea anemones originated in the Coral Triangle region, followed by an independent geographical radiation in the Western Indian Ocean. Thus,** **distribution and abundance of** **anemone** **fish** **are** **intrinsically linked to the** **presence** **and abundance of** **giant** **sea anemones** **^135^** **^,^** **^136^** **. Anemonefish-host anemones are, in turn, exclusively found in** **the** **tropical Indo-Pacific Ocean** **,** **with no presence in the Eastern Pacific nor Caribbean regions.** **^26^** **^,^** **^32^** **^”^**

 3. Five paragraph. Not sure what you mean by “consequently fixed by natural selection” - do you mean that the inversion is always fixed different (one arrangement at frequency 1.0 in one species, and the other arrangement fixed at 1.0 in the other species? One alternative would be that the inversion is polymorphic in both species, so this need to be clarified. What is here referred to as “supergenes” is also somewhat unclear. Usually, the definition of a supergene is that is e.g. an inversion (or some other low-recombination region) which remains polymorphic within populations - and so this does not match the statement of “fixed by selection”....

**The reviewer’s comment raises a valid point. This has been clarified so it now reads: “** **This** **persistence might be attributed to** **genomic inversions that** **disrupt recombination** **and creat** **e** **clusters of loci controlling ecologically important traits that** **may** **consequently** **be** **fixed by natural selection** **or through genetic drift** **.** **”**

 Under 4.3. One obvious question after the presentation of the extremely long life-span is, does anemone fish have undetermined growth? That is, do they grow to large for their anemone, and so have to move to another and larger anemone? (Also, are the anemones also similarly long-lived?)

**As suggested by the reviewer, we have now discussed this. The last paragraph of this section reads: “** **After exploring the remarkable long lifespan of anemonefish, a few other compelling questions arise: Do anemonefish have undetermined growth, growing too large for their host sea anemone and eventually having to move to another and larger anemone? Does the longevity of the host sea anemones parallel that of anemonefish? In a groundbreaking milestone, Rueger and colleagues (2022) provided the first-ever experimental evidence for the first question. Their study sheds light on the remarkable growth plasticity of anemonefish in response to their mutualistic interaction with sea anemones, that is, anemonefish adjust their growth rate to make sure they are the ideal size for their hosts. Thus, emphasizing the crucial role of mutualisms in shaping species’ adaptations and ecological relationships. Moreover, this research opens up exciting new avenues for exploring the underlying molecular mechanisms behind this phenomenon: What are the cues necessary for the fish to decide how large it needs to be? Having explored the growth plasticity of anemonefish, we now shift the focus now to the longevity of the host sea anemone and its intricate association with the presence and size of their anemonefish counterparts. Various studies have shown that giant sea anemones are short lived compared to their fish symbionts, with some species like *E. quadricolor* and *H. crispa* having turnover times of only** **3–5 years** **.** **The short lifespans of these host anemones** **certainly** **affect their resident anemonefish** **as they** **need to migrate among hosts during their lifetimes, and do so when space becomes available nearby** **. As a result,** **some anemonefish may** **rely on** **the presence of multiple anemone hosts within a** **relatively small** **area** **of reef, leading to potential constraints on their** **lifespans due to host turnover** **. This is particularly evident in** **areas with low and declining host abundance** **.”**

 Under 4.3, second paragraph. What is the difference between “lifespan” and “longevity” in the next last sentence of this paragraph?

**The reviewer is right, and we have clarified this in the first paragraph so it now reads: “** **The evolutionary theory of aging predicts that individuals with low extrinsic mortality will show delayed senescence (i.e., the process of physiological deterioration with age) and increased lifespan (reviewed in Ref.** **194** **).** **It is important to note that although the terms “lifespan” and “longevity” may sound interchangeable, they hold distinct meanings when discussing life expectancy. Lifespan refers to the maximum potential duration of life for a given species or population. Longevity, on the other hand, describes the ability to live a long life beyond the species-specific average age at death (reviewed in Refs. ). To illustrate, if the average lifespan of a species is 10 years, for example, it means that, on average, individuals of that species are expected to live 10 years. However, if the longevity of the same species is 10 years, then it means that some individuals may live up to 10 years, but the average lifespan may still be much lower than 10 years.”**

 Under 6. First sentence - please spell out which are these fundamental questions you are referring to.

**We have added this, it now reads: “** **Anemonefish have become an invaluable model system for answering some of the most fundamental and long-standing questions in evolutionary genomics.** **How do speciation events occur, and what are the underlying genetic mechanisms driving this process? What genetic changes underlie the development of morphological, physiological, and behavioral traits? How do organisms adapt to their environments, and what role does natural selection play in shaping their genomic architecture? How can we integrate genomic data from multiple species to gain insights into the evolutionary processes shaping biodiversity?”**

 That the fish never abandon their anemone is a very interesting fact that should perhaps be highlighted earlier (or I did miss it when reading), together with the very long lifespan, to illustrate what is the unique features of the anemonefish as a model.

**As suggested by the reviewer, we have now included this in section 2.1 “Practical features of anemonefish for experimentation”.**

# Reviewer report: Reviewer response for version 1

**DOI:** 10.5256/f1000research.143530.r164241

**Published:** 2023-04-13

**Authors:** Fabio Cortesi

**Affiliations:** 1 School of the Environment and Queensland Brain Institute, The University of Queensland, Brisbane, QLD, Australia

Herrera and colleagues review the current standings in anemonefish multi-omics research in their article. They propose that anemonefish are an ideal model for studying ecological, evolutionary and developmental processes because they show traits shared across teleosts and vertebrates and many unique features that set them apart. Especially their obligate relationship with a host anemone and a hierarchy-dependent hermaphroditic lifestyle makes them prime candidates to study processes such as adaptive radiations, mutualism, social dynamics, developmental plasticity, and adaptation to climate change, including ocean warming and acidification. Indeed, in recent years, the anemonefish research community has grown considerably. There are now multiple labs around the globe that use the latest ‘omic’ technologies (e.g., long-read genome sequencing, proteomics, transcriptomics and CRISPR/Cas9) combined with sophisticated imaging and behavioural approaches to study the life histories of these fascinating critters.

 I really enjoyed reading this review as it provides both a historical account of the progress made in using molecular approaches and a current-day account and outlook of where the study of anemonefish biology will likely lead us. The review does an excellent job of incorporating the latest studies in the anemonefish ‘omic’ space without prioritising one field or study over another. I also found the article flow easy to follow, and the figures were well-designed and informative. Certainly, I gained several new insights and research ideas from reading this review.

 I only have a few minor comments I recommend addressing before the indexing of the article in F1000 should occur.  

 Minor comments:

 Figure 1

 Zoomed-in panel: What are the multiple looping arrows for the proteome circle referring to? One of them is labelled with post-translational modifications, which makes sense. What are the other two referring to?

 Main panel: In the main text, you say that the first chromosome-scale genome was released in 2018, but here it is shown as 2019. If I remember correctly, the study was first published in pre-print in 2018 and then after peer review in 2019. I recommend sticking to one or the other.

 2. Anemonefish as a model system for evolutionary biology

 Change ‘Furthermore, the fish serves as a supplemental […].’ to ‘Furthermore, the fish provides supplemental nutrition […].'

 In the sentence: 'Distribution varies greatly for each species, some are widespread (e.g., A. clarkii, A. sandaracinos), while others have a limited regional distribution (e.g., A. bicinctus, A. percula) or are even restricted to a few islands (e.g., A. chagosensis, A. latezonatus).'

 A. latezonatus occurs along the subtropical coast of Eastern Australia and is not restricted to a few islands. Did you mean A. mccullochi instead?

 Change ‘Constructing a high-quality chromosome-level assembly for a species with no previous genome-scale data was certainly a major achievement in a world of sticklebacks and zebrafish’ to ‘Constructing a high-quality chromosome-level assembly for a species with no previous genome-scale data was certainly a major achievement in a world dominated by stickleback and zebrafish genomic research’.

 2.3 Insights from comparative transcriptomics

 The first two sentences talk about the initial molecular work in anemonefish. I recommend adding early-day studies about the population genetics of these fishes here, e.g., Jeff Jones and colleagues’ seminal work using microsatellites to show that anemonefish settle close to home was published in the early 2000s.

 2.4 The rise of proteomics

 I recommend splitting this very long sentence into two: ‘Proteomic techniques have been classified as shotgun, the optimal method for discovering more proteins but with the drawback that has reduced quantitative accuracy and reproducibility, or targeted, which is better for reproducibility if the proteins in question are known but limited in the number of measurements and therefore the number of peptides that can be identified.’

 Add the verb ‘using’ to the following sentence ‘SWATH-MS is versatile and has been used […].’

 3.3 Phenotypic plasticity and genetic assimilation in development and evolution of anemonefish

 I suggest rewriting the following sentence to improve clarity: ‘Species with adult individuals that can be experimentally induced to transition between distinct phenotypes are notably valuable as they make it possible to isolate phenotypic effects of gene expression by comparing the gene expression profiles of groups of individuals who differ in their phenotypes due to plasticity rather than genetic differences.’

 How can these species be leveraged to study plastic versus genetic effects on a phenotype?

 4.1 Sex change

 The authors do a great job of describing the transcriptomic research that has taken place to investigate sex change in anemonefish, noting that a brain-to-gonad axis exists in these fishes. However, the timing and speed at which these changes occur still need to be debated, and some mention of this should ideally be incorporated here. For example, in addition to the relatively fast changes observed by Casas and colleagues, recent work from the Rhodes lab shows that very long periods can also occur between the feminisation of the brain and the gonads (e.g., Active feminization of the preoptic area occurs independently of the gonads in Amphiprion ocellaris).

 4.2 Pigmentation and color patterns

 I would like to point the authors to a recent study of ours that investigated the role of white stripes in the antagonistic behaviour of anemonefishes: ‘Mitchell et al.., 2023 Higher ultraviolet skin reflectance signals submissiveness in the anemonefish, Amphiprion akindynos. Behavioral Ecology’.

 While I am usually against pushing our work onto the authors of an article, in this case, this study addresses one of the main points raised by the authors, which is that we provide behavioural evidence for: ‘Young recruits are colored distinctly different than older juveniles to avoid antagonistic and aggressive behaviors from the larger individuals’.

 I recommend changing the second sentence here: ‘Morphotypes such as albinism or individuals with no bands in species that usually have, are never or very rarely observed in the wild but can be found in the aquarium trade industry (reviewed in Ref. 16). In the wild, mutations that result in such drastic color pattern alterations have a negative effect on the survival of individuals and are therefore negatively selected against, but they can be bred for several generations in aquaculture.’

 I would be more speculative about the effects of selection, i.e., say something like ‘are likely to have a negative effect’. Anemonefish without white bands have evolved from a (multi-)banded ancestor in nature. Hence selection favoured these extreme phenotypes under certain circumstances.

 4.3 Longevity and lifespan

 In line with the original article, change the following statement to be damselfish specific as plenty of bigger coral reef fishes live beyond 30 years: ‘Noteworthy, this estimate is two times greater than the longevity estimated for any other coral reef fish’.

Is the review written in accessible language?

Yes

Are all factual statements correct and adequately supported by citations?

Yes

Are the conclusions drawn appropriate in the context of the current research literature?

Yes

Is the topic of the review discussed comprehensively in the context of the current literature?

Yes

Reviewer Expertise:

Evolution, neuroethology, marine biology, ichthyology, sensory biology, molecular ecology, behavioural ecology, genomics, transcriptomics

I confirm that I have read this submission and believe that I have an appropriate level of expertise to confirm that it is of an acceptable scientific standard, however I have significant reservations, as outlined above.

## References

[1] : Higher ultraviolet skin reflectance signals submissiveness in the anemonefish, Amphiprion akindynos. *Behav Ecol* .2023;34(1) : 10.1093/beheco/arac089 19-32 10.1093/beheco/arac089 36789393PMC9918861

# Response

**Published:** 2023-08-03

**Authors:** Marcela Herrera Sarrias

**Affiliations:** Okinawa Institute of Science and Technology, Japan

Minor comments:

 Figure 1

 Zoomed-in panel: What are the multiple looping arrows for the proteome circle referring to? One of them is labelled with post-translational modifications, which makes sense. What are the other two referring to?

**The reviewer is right, and we have modified the figure for clarification. All loop arrows for all circles have been labelled.**

 Main panel: In the main text, you say that the first chromosome-scale genome was released in 2018, but here it is shown as 2019. If I remember correctly, the study was first published in pre-print in 2018 and then after peer review in 2019. I recommend sticking to one or the other.

**The reviewer is right, and we apologize for the confusion. We have corrected this so it now reads: “** **The year 201** **9** **saw the publication of the first chromosome-scale genome for an anemonefish ^4 ^(** **Figure 1)** **.** **”**

 2. Anemonefish as a model system for evolutionary biology

 Change ‘Furthermore, the fish serves as a supplemental […].’ to ‘Furthermore, the fish provides supplemental nutrition […].'

**This has been modified as suggested by the reviewer.**

 In the sentence: 'Distribution varies greatly for each species, some are widespread (e.g., A. clarkii, A. sandaracinos), while others have a limited regional distribution (e.g., A. bicinctus, A. percula) or are even restricted to a few islands (e.g., A. chagosensis, A. latezonatus).'

 A. latezonatus occurs along the subtropical coast of Eastern Australia and is not restricted to a few islands. Did you mean A. mccullochi instead?

**The reviewer is right. We have replaced *A. latezonatus* for *A. mccullochi*.**

 Change ‘Constructing a high-quality chromosome-level assembly for a species with no previous genome-scale data was certainly a major achievement in a world of sticklebacks and zebrafish’ to ‘Constructing a high-quality chromosome-level assembly for a species with no previous genome-scale data was certainly a major achievement in a world dominated by stickleback and zebrafish genomic research’.

**This has been changed as suggested by the reviewer.**

 2.3 Insights from comparative transcriptomics

 The first two sentences talk about the initial molecular work in anemonefish. I recommend adding early-day studies about the population genetics of these fishes here, e.g., Jeff Jones and colleagues’ seminal work using microsatellites to show that anemonefish settle close to home was published in the early 2000s.

**This has been changed as suggested by the reviewer, so it now reads: “** **The first molecular insights of anemonefish biology came well before any of the genomes now available were sequenced.** **Early-day research by Jones and colleagues with microsatellites shed a light on the population genetics and dispersal patterns of anemonefish in Kimbe Bay, Papua New Guinea. Yet, it was a 2010** **study using quantitative polymerase chain reaction (qPCR) to investigate the role of the aromatase *cyp19a1* gene on sex differentiation of the yellowtail clownfish *A. clarkii*** **^74^** **that really p** **ioneer** **ed molecular** **research in anemonefish.** **”**

 2.4 The rise of proteomics

 I recommend splitting this very long sentence into two: ‘Proteomic techniques have been classified as shotgun, the optimal method for discovering more proteins but with the drawback that has reduced quantitative accuracy and reproducibility, or targeted, which is better for reproducibility if the proteins in question are known but limited in the number of measurements and therefore the number of peptides that can be identified.’

**This has been rewritten so it now reads: “** **Proteomic techniques have been classified** **into two categories:** **shotgun** **and targeted. Shotgun** **is** **the optimal method for discovering more proteins** **despite its** **drawback** **of** **reduced quantitative accuracy and reproducibility** **. On the other hand,** **targeted** **techniques** **are** **better for reproducibility** **when** **the proteins in question are known but** **are** **limited in the number of measurements and therefore the number of peptides that can be identified.** **”**

 Add the verb ‘using’ to the following sentence ‘SWATH-MS is versatile and has been used […].’

**This has been changed as suggested by the reviewer.**

 3.3 Phenotypic plasticity and genetic assimilation in development and evolution of anemonefish

 I suggest rewriting the following sentence to improve clarity: ‘Species with adult individuals that can be experimentally induced to transition between distinct phenotypes are notably valuable as they make it possible to isolate phenotypic effects of gene expression by comparing the gene expression profiles of groups of individuals who differ in their phenotypes due to plasticity rather than genetic differences.’

 How can these species be leveraged to study plastic versus genetic effects on a phenotype?

**This has been rewritten so it now reads: “** **Species with adult individuals that can be experimentally induced to transition between distinct phenotypes are** **highly** **valuable** **.** **T** **hey make it possible to isolate phenotypic effects of gene expression by comparing the gene expression profiles of groups of individuals who differ in their phenotypes due to plasticity rather than genetic differences.** **”**

 4.1 Sex change

 The authors do a great job of describing the transcriptomic research that has taken place to investigate sex change in anemonefish, noting that a brain-to-gonad axis exists in these fishes. However, the timing and speed at which these changes occur still need to be debated, and some mention of this should ideally be incorporated here. For example, in addition to the relatively fast changes observed by Casas and colleagues, recent work from the Rhodes lab shows that very long periods can also occur between the feminisation of the brain and the gonads (e.g., Active feminization of the preoptic area occurs independently of the gonads in Amphiprion ocellaris).

**As suggested by the reviewer this has been added: “The feminization of the brain in anemonefish is inarguably an active process, and the timing and speed at which these changes occur remain a compelling and active field of research. The process spans a wide time frame, with brain expression profiles changing relatively rapidly after female removal (0 to 11 days in males and 15 to 30 days in transitional males** **^12^** **) and complete gonadal changes taking much longer (sometimes over the course of several years). Altogether, anemonefish provide a unique opportunity to explore the molecular, biochemical, and physiological mechanisms underlying sex change in vertebrates.”**

 4.2 Pigmentation and color patterns

 I would like to point the authors to a recent study of ours that investigated the role of white stripes in the antagonistic behaviour of anemonefishes: ‘Mitchell et al.., 2023 Higher ultraviolet skin reflectance signals submissiveness in the anemonefish, Amphiprion akindynos. Behavioral Ecology’.

 While I am usually against pushing our work onto the authors of an article, in this case, this study addresses one of the main points raised by the authors, which is that we provide behavioural evidence for: ‘Young recruits are colored distinctly different than older juveniles to avoid antagonistic and aggressive behaviors from the larger individuals’.

**As suggested by the reviewer this has been changed so it now reads:** **“** **Young recruits are colored distinctly different than older juveniles to potentially avoid antagonistic and aggressive behaviors from the larger individuals.** **^175^** **Loss of white vertical bars during ontogeny has indeed been observed in multiple *Amphiprion* species.** **^14^** **Mitchell and colleagues (2023) further showed that UV reflectance in anemonefish (from their orange and white bars) has a functional role in modulating aggression and signaling submissiveness in family groups.”**

 I recommend changing the second sentence here: ‘Morphotypes such as albinism or individuals with no bands in species that usually have, are never or very rarely observed in the wild but can be found in the aquarium trade industry (reviewed in Ref. 16). In the wild, mutations that result in such drastic color pattern alterations have a negative effect on the survival of individuals and are therefore negatively selected against, but they can be bred for several generations in aquaculture.’

 I would be more speculative about the effects of selection, i.e., say something like ‘are likely to have a negative effect’. Anemonefish without white bands have evolved from a (multi-)banded ancestor in nature. Hence selection favoured these extreme phenotypes under certain circumstances.

**This has been changed so it now reads: “** **In the wild, mutations that result in such drastic color pattern alterations** **are likely to** **have a negative effect on the survival of individuals and are therefore negatively selected against, but they can be bred for several generations in aquaculture.** **”**

 4.3 Longevity and lifespan

 In line with the original article, change the following statement to be damselfish specific as plenty of bigger coral reef fishes live beyond 30 years: ‘Noteworthy, this estimate is two times greater than the longevity estimated for any other coral reef fish’.

**The reviewer is right, and this has been changed so it now reads “… longevity estimated for any other pomacentrid and up to two times …”**
